# The HDAC inhibitor zabadinostat is a systemic regulator of adaptive immunity

**DOI:** 10.1038/s42003-023-04485-y

**Published:** 2023-01-26

**Authors:** Geng Liu, Wojciech Barczak, Lian Ni Lee, Amit Shrestha, Nicholas M. Provine, Gulsah Albayrak, Hong Zhu, Claire Hutchings, Paul Klenerman, Nicholas B. La Thangue

**Affiliations:** 1 Laboratory of Cancer Biology, Department of Oncology, University of Oxford, Old Road Campus Research Building, Oxford, OX3 7DQ UK; 2 Peter Medawar Building for Pathogen Research, University of Oxford, Oxford, OX1 3SY UK; 3 Translational Gastroenterology Unit, Nuffield Department of Medicine, University of Oxford, Oxford, OX3 9DU UK; 4 Department of Medical Oncology, Cancer Center, West China Hospital, Sichuan University, 610041 Chengdu, China; 5 Celleron Therapeutics Ltd, Magdalen Centre, Oxford Science Park, Oxford, OX4 4GA UK

**Keywords:** Adjuvants, Translational immunology

## Abstract

Protein acetylation plays a key role in regulating cellular processes and is subject to aberrant control in diverse pathologies. Although histone deacetylase (HDAC) inhibitors are approved drugs for certain cancers, it is not known whether they can be deployed in other therapeutic contexts. We have explored the clinical HDAC inhibitor, zabadinostat/CXD101, and found that it is a stand-alone regulator of the adaptive immune response. Zabadinostat treatment increased expression of MHC class I and II genes in a variety of cells, including dendritic cells (DCs) and healthy tissue. Remarkably, zabadinostat enhanced the activity of DCs, and CD4 and CD8 T lymphocytes. Using an antigenic peptide presented to the immune system by MHC class I, zabadinostat caused an increase in antigen-specific CD8 T lymphocytes. Further, mice immunised with covid19 spike protein and treated with zabadinostat exhibit enhanced covid19 neutralising antibodies and an increased level of T lymphocytes. The enhanced humoral response reflected increased activity of T follicular helper (Tfh) cells and germinal centre (GC) B cells. Our results argue strongly that zabadinostat has potential to augment diverse therapeutic agents that act through the immune system.

## Abstract (web summary)

The clinical HDAC inhibitor zabadinostat increases MHC class I and II expression in dendritic cells, activates T and B cells, and enhances adaptive immune responses to COVID-19 spike protein in mice.

## Introduction

Lysine acetylation is a key post-translational modification of proteins that regulates many cellular processes^1,2^. Although historically regarded as a mechanism connected with the control of chromatin activity, it is now known to have much wider relevance in cell biology; in fact some studies suggest that up to 50% of cellular proteins are subject to control though lysine acetylation^3^. Two families of enzymes control the deposition of the acetyl group on the lysine side chain; histone acetyl-transferases (HATs) mediate the acetylation event, and histone deacetylase (HDAC) regulates its removal^1^. Both HDAC and HAT enzymes are frequently under abnormal control in cancer^4^. Consequently, a variety of approaches have been taken to develop drugs that inhibit HDAC activity and thereby reinstate normal acetylation control. Surprisingly, however, the therapeutic intervention of HDACs has met with limited clinical success and although a few drugs have been approved, they are registered for use in relatively minor haematological malignancies^5,6^. It is possible therefore that there is further scope to deploy HDAC inhibitors in other clinical contexts.

With this idea in mind, we have explored the HDAC inhibitor zabadinostat (also known as CXD101), with the objective of assessing whether the drug can be deployed in clinical situations other than cancer. Zabadinostat is a promising second-generation HDAC inhibitor undergoing clinical trials with selective activity towards class 1 HDAC subunits^7,8^. It is a potent anti-proliferative agent on cancer cells and in human clinical studies has demonstrated a favourable safety profile, with subsequent studies documenting encouraging durable disease inhibition in late-stage cancer^7,9^. In combination with immune checkpoint inhibitors, like anti-PD-1, zabadinostat exhibits remarkable synergy in murine tumour progression models^10^. The mechanism responsible may be related to the increased MHC class I and II gene expression which occurs in treated cancer cells^10^. The increased level of MHC gene expression seen in murine tumours reflected a marked impact on tumour-infiltrating lymphocytes and other immune-relevant cells in the tumour micro-environment^10^, arguing strongly that the increased MHC expression fosters renewed engagement of the immune response.

Here, we have investigated whether zabadinostat has the ability to regulate the immune response in healthy animals. Our results show that zabadinostat treatment causes a sustained and significant increase in MHC gene expression in cells and tissue, together with enhanced CD4 and CD8 T lymphocyte, and B-cell activity. In mice immunised with an experimental MHC class I specific antigenic peptide recognised by CD8 T lymphocytes, treatment with zabadinostat caused an increase in the level of antigen-specific CD8 T lymphocytes. Most significantly, in mice immunised with covid19 spike S1 protein, treatment with zabadinostat enhanced the neutralising antibody response which blocked spike S1 protein binding to its ACE2 receptor. The enhanced humoral response reflects the increased activity of T-follicular helper (Tfh) and germinal centre (GC) B cells upon zabadinostat treatment. The ability of zabadinostat to act systemically on the immune response defines a new application and a powerful rationale for extending its clinical application.

## Results

### Zabadinostat treatment causes increased MHC gene expression in diverse types of cells, including epithelial cells

**Fig. 1:**
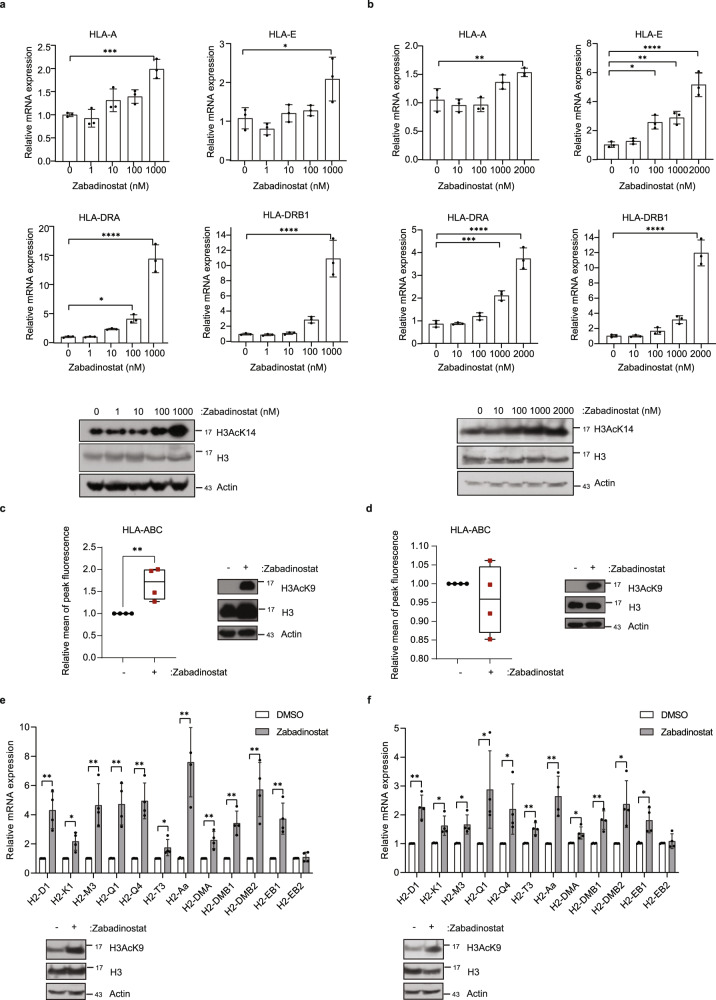
Expression of MHC class I and class II genes upon zabadinostat treatment. **a** Quantitative reverse transcription PCR (qRT-PCR) of MHC class I and class II genes in BEAS2B cells treated for 3 days with 1, 10, 100, 1000 nM zabadinostat or DMSO control; *n* = 3; results presented as mean values +/−SD; one-way ANOVA; The acetylation mark (H3AcK14) was detected by immunoblotting. **b** Quantitative reverse transcription PCR (qRT-PCR) of MHC class I and class II genes in NBE1 cells treated for 3 days with 10, 100, 1000, 2000 nM zabadinostat or DMSO control; *n* = 3; results presented as mean values +/−SD; one-way ANOVA. The acetylation mark (H3AcK14) was detected by immunoblotting. **c** Flow cytometry analysis of extracellular HLA class I proteins in BEAS2B cells treated for 2 days with 1 µM zabadinostat or DMSO control, *n* = 4, results presented as mean values +/−SD, Student’s *t* test; The acetylation mark (H3AcK9) was detected by immunoblotting in BEAS2B cells. **d** Flow cytometry analysis of extracellular HLA class I proteins in NBE1 cells treated for 2 days with 1 µM zabadinostat or DMSO control, *n* = 4, results presented as mean values +/−SD, Student’s *t* test; The acetylation mark (H3AcK9) was detected by immunoblotting in NBE1 cells; Quantitative reverse transcription PCR (qRT-PCR) of MHC class I and class II genes in lung RNA from Balb/c mice treated for 14 days (**e**) and 30 days (**f**) with 10 mg/kg zabadinostat or DMSO control; *n* = 4; results presented as mean values +/−SD; one-way ANOVA. The acetylation mark (H3AcK9) of representative mouse samples was detected by immunoblotting.

In previous studies, we described the ability of zabadinostat treatment on a variety of human cancer cell lines to increase the expression of MHC class I and class II genes^10^. We continued these studies by analysing the properties of zabadinostat in non-cancer cell lines and normal mouse tissue to determine whether the induction of MHC gene expression was apparent in non-pathological conditions. We chose to study BEAS2B and NBE1 cells, cell lines derived from normal human lung bronchial epithelium^11,12^. We titrated the level of zabadinostat on the cells and measured MHC class I and II gene expression by qPCR after 3 days. We observed a titratable increase in the expression of MHC class I and II genes; in BEAS2B cells, *HLA-A, -C, -E, -DMB, -DOA, -DOB, -DPB1, -DRA* and -*DRB1* genes increased (Fig. 1a and Supplementary Fig. 1a) whereas in NBE1 cells, *HLA-A, -E, -DMB, -DOA, -DRA* and *-DRB1* genes increased (Fig. 1b and Supplementary Fig. 1b). The change in MHC class I gene expression reflected a similar increase in protein level measured by flow cytometry in BEAS2B and NBE1 cells (Fig. 1c, d and Supplementary Fig. 2) which also coincided with an increased level of histone H3 lysine acetylation in cells treated with zabadinostat (Fig. 1a–d). Similar effects on MHC class I expression and protein level were seen in Jurkat, CEM and Raji cells (Supplementary Figs. 2 and 3a–f); minimal effects on proliferation and cytotoxicity were observed in all cell lines studied (Supplementary Fig. 1c, d).

We next evaluated whether a similar effect of zabadinostat was observed in lung tissue of treated mice. For this, we used normal Balb/c mice that were treated with two cycles of zabadinostat (10 mg/kg), which is an established well-tolerated dosing regimen in mice with no observable side effects^10^. Lung tissue was harvested from the treated and control mice at day 14 and 30 post treatment, and the expression of MHC H2 class I and class II genes was measured. We observed a significant increase in the expression of the MHC class I genes *H2-D1, -K1, -M3, -Q1, -Q4* and *-T3* together with elevated expression of MHC class II genes *H2-Aa, -DMA, -DMB1, -DMB2* and -*EB1* (Fig. 1e, f). We conclude therefore that zabadinostat increases tissue expression of MHC class I and II genes when administered to animals.

**Fig. 2:**
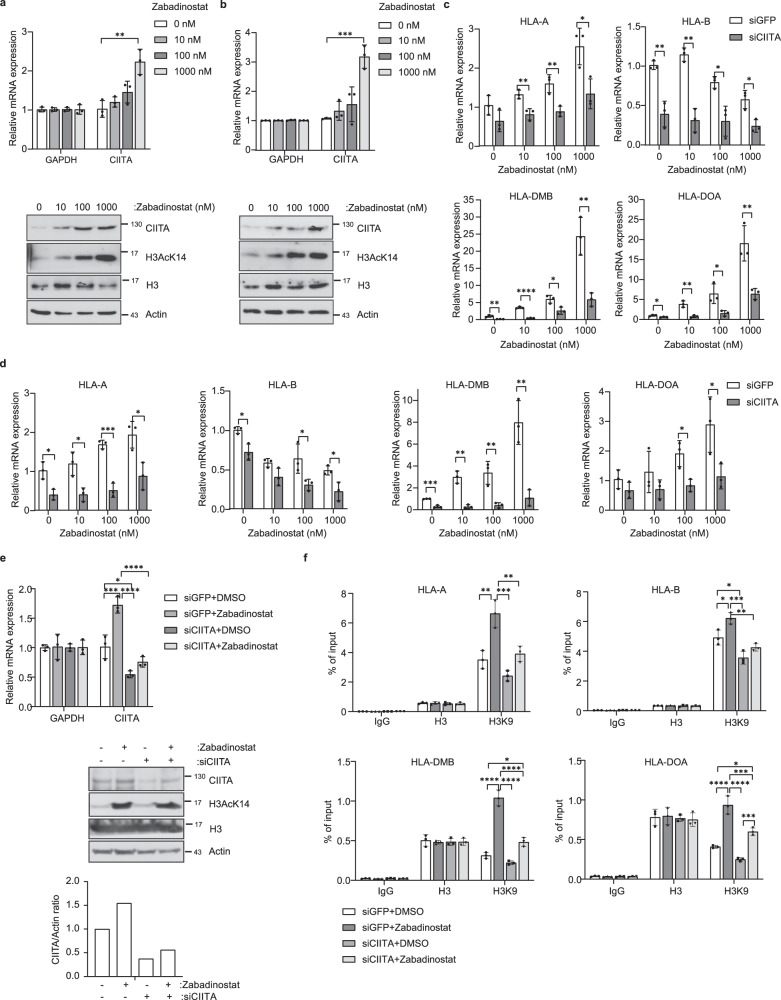
The effect of CIITA on the expression of MHC class I and class II genes. **a** Quantitative reverse transcription PCR (qRT-PCR) of CIITA in BEAS2B cells treated for 3 days with 10, 100, 1000 nM zabadinostat or DMSO control; *n* = 3; results presented as mean values +/−SD; one-way ANOVA; The CIITA protein level and acetylation mark (H3AcK14) was detected by immunoblotting. **b** Quantitative reverse transcription PCR (qRT-PCR) of CIITA in NBE1 cells treated for 3 days with 10, 100, 1000 nM zabadinostat or DMSO control; *n* = 3; results presented as mean values +/−SD; one-way ANOVA; The CIITA protein level and acetylation mark (H3AcK14) was detected by immunoblotting. **c** Quantitative reverse transcription PCR (qRT-PCR) of MHC genes in BEAS2B cells treated for 2 days with 50 nM siCIITA and for 3 days with 10, 100, 1000 nM zabadinostat or DMSO control; *n* = 3; results presented as mean values +/−SD; one-way ANOVA. **d** Quantitative reverse transcription PCR (qRT-PCR) of MHC class I genes in NBE1 cells treated for 2 days with 50 nM siCIITA and for 3 days with 10, 100, 1000 nM zabadinostat or DMSO control; *n* = 3; results presented as mean values +/−SD; one-way ANOVA. **e** Quantitative reverse transcription PCR (qRT-PCR) of CIITA in BEAS2B cells treated for 2 days with 50 nM siCIITA and for 3 days with 10, 100, 1000 nM zabadinostat or DMSO control; *n* = 3; results presented as mean values +/−SD; one-way ANOVA. The CIITA protein level and acetylation mark (H3AcK14) were detected by immunoblotting and quantified. **f** Histone H3 and H3AcK9 ChIP on MHC gene promoters in BEAS2B cells treated for 2 days with 50 nM siCIITA and for 3 days with 1000 nM zabadinostat or DMSO control; *n* = 3; results presented as mean values +/−SD; one-way ANOVA.

We progressed on to evaluate the mechanisms that are responsible for the increased expression of MHC genes. We hypothesised that genes involved with transcriptional control of the MHC loci might be involved, and focussed our attention on the *CIITA* gene, a transcriptional co-activator protein which regulates MHC gene expression^13^. Interestingly, *CIITA* expression and protein levels increased upon zabadinostat treatment; for example, in NBE1 and BEAS2B cells, in addition to the cancer cell line HCT116 (Fig. 2a, b and Supplementary Fig. 4a, b). We then addressed the role of *CIITA* in the expression of MHC genes by introducing siRNA into cells and evaluating any impact on expression; the CIITA siRNA reduced the expression of the target RNA (Supplementary Figs. 4c and 5a, b). In zabadinostat-treated BEAS2B cells, siCIITA reduced the level of MHC class I *HLA-A* and -*B* but had less effect on *HLA-C* and *-E* (Fig. 2c and Supplementary Fig. 5c); note that the residual activation of *HLA-A* in CIITA siRNA treated cells (Fig. 2c) may reflect incomplete depletion of *CIITA*. There were striking effects on the expression of MHC class II *HLA-DMB, -DOA, -DOB, -DRA* and -*DRB1,* whereas *HLA-DPB1* did not show reduced expression (Fig. 2c and Supplementary Fig. 5c). Similar results were seen in NBE1 cells, which showed that CIITA siRNA reduced the level of MHC class I *HLA-A, -B* and *-C* but not *-E* (Fig. 2d and Supplementary Fig. 5d). There were also significant effects on the expression of MHC class II *HLA-DMB*, *-DOA* and *-DOB* whereas *HLA-DPB1, -DRA* and -*DRB1* were hardly affected (Fig. 2d and Supplementary Fig. 5d). In zabadinostat-treated HCT116 cancer cells, treatment with the CIITA siRNA reduced the level of MHC class I *HLA-A* and -*C* but had less effect on *HLA-B* and *-E* (Supplementary Fig. 4d). Similarly, there were striking effects on the expression of MHC class II *HLA-DMB, -DOA, -DOB, -DPB1* and -*DRA*, whereas *HLA-DRB1* was hardly affected (Supplementary Fig. 4d).

Next, we assessed the mechanism through which *CIITA* affected MHC gene expression. We used ChIP to measure the impact on the acetylation of histone (H) 3 lysine (K) 9 across the promoter region of MHC genes in BEAS2B cells, as H3K9 acetylation coincides with transcriptional activity (Fig. 2e, f)^1^. Zabadinostat treatment increased the level of the acetylation mark at H3K9 compared to the control untreated cells, without any significant change in the level of total H3 bound to the MHC gene promoters (Fig. 2f and Supplementary Fig. 5e). In conditions of reduced *CIITA* (Fig. 2e), the increased level of H3K9 acetylation upon zabadinostat treatment was, for relevant genes such as *HLA-A* and *HLA-DMB*, less marked compared to the control siRNA treatment (Fig. 2f and Supplementary Fig. 5e). Furthermore, there was a relationship between the effect of CIITA siRNA on transcription and the level of H3K9 acetylation. For example, *HLA-A* gene expression was reduced reflecting a similar reduction in ChIP signal for acetylated H3K9 upon CIITA siRNA treatment (Fig. 2c, f) compared to *HLA-C*, where a limited effect on transcription of CIITA siRNA treatment reflected a minimal change in ChIP activity upon CIITA siRNA treatment (Supplementary Fig. 5c, e). Overall, these results suggest that *CIITA* is a positive regulator of MHC class I and II genes in zabadinostat-treated cells and provide a plausible mechanism that this occurs through increased acetylation.

### Zabadinostat augments MHC expression in dendritic cells, and activates T and B lymphocytes

**Fig. 3:**
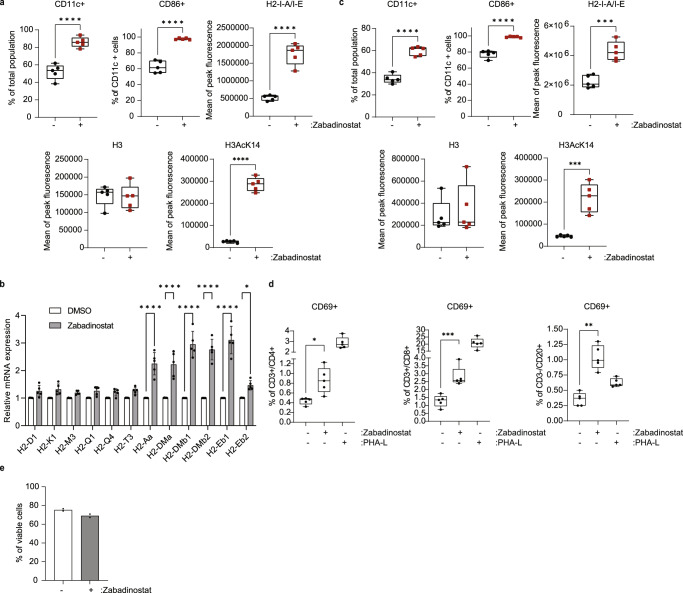
Zabadinostat augments MHC expression in dendritic cells, and activates T and B lymphocytes. **a** Flow cytometry analysis of the extracellular MHC class II protein level in bone marrow (collected from C57BL/6)-derived dendritic cells (CD11c+/CD86+) treated with 1 µM zabadinostat or DMSO control for 48 h; *n* = 5, Student’s *t* test; the acetylation mark (H3AcK14) and total histone 3 level were detected by flow cytometry; *n* = 5, Student’s *t* test. **b** Quantitative reverse transcription PCR (qRT-PCR) of MHC class I and II genes was also performed; *n* = 5; results presented as mean values +/−SD; one-way ANOVA. **c** Flow cytometry analysis of the extracellular MHC class II protein level in bone marrow (collected from Balb/c)-derived dendritic cells (CD11c+/CD86+) treated with 1 µM zabadinostat or DMSO control for 48 h, *n* = 5; the acetylation mark (H3AcK14) and total histone 3 level were detected by flow cytometry; *n* = 5; Student’s *t* test. **d** Activation of CD4, CD8 T cells and B cells upon 1 µM zabadinostat treatment or DMSO control was evaluated in splenocytes collected from Balb/c mice; *n* = 5; one-way ANOVA. **e** Viability of splenocytes measured by flow cytometry with L/D staining; graph represents pooled results from two animal experiments (see Fig. 5 (*n* = 4) and Supplementary Fig. 15 (*n* = 4)); results presented as mean values +/−SD.

We were interested to explore the effect of zabadinostat treatment on antigen-presenting cells and for this part of the study used bone marrow-derived DCs taken from C57BL/6 and Balb/c mice. We measured CD11c as the DC marker, and CD86 for the DC maturation and activation marker^14,15^. Upon zabadinostat treatment, there was a highly significant increase in the size of the CD11c and CD86 DC populations, which coincided with increased H3K14 acetylation (Fig. 3a, b and Supplementary Fig. 6). Under the treatment conditions, there was a significant increase in MHC class II expression in DCs derived from both strains of mice (Fig. 3a–c); MHC class I levels underwent minimal change upon treatment (Supplementary Fig. 3g). We conclude therefore that zabadinostat augments DC maturation, which coincides with increased MHC class II gene expression.

We performed a similar analysis in CD4 and CD8 T cells, and B cells. Upon zabadinostat treatment, there was a significant increase in the level of CD69, which we used as a marker for activated lymphocytes (Fig. 3d and Supplementary Fig. 7^16^). It is noteworthy that under these experimental conditions, there was no loss of cell viability upon zabadinostat treatment (Fig. 3e). These results indicate that zabadinostat increases the level of activated T and B lymphocytes.

### Zabadinostat augments the antigen-specific CD8 T-cell response

**Fig. 4:**
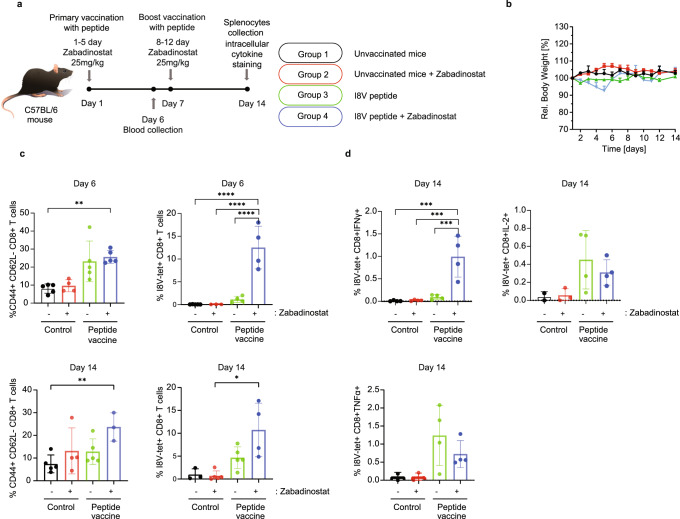
Zabadinostat enhances the CD8 T-cell response. **a** Schematic representation of the immunogenicity experiment with zabadinostat and peptide vaccine. C57BL/6 mice were treated with orally administrated zabadinostat at 25 mg/kg for 14 days (5 days on/2 days off) and intravenous I8V peptide (days 1 and 7) with respect to vehicle-only control; *n* = 5 per group; **b** relative body weight representation of treated and non-treated mice; *n* = 5; **c** general effector memory T-cell activation (CD8 + CD44 + CD62L− positive T cells) at days 6 (in the blood) and 14 (in splenocytes), I8V-peptide-specific CD8-positive T cells stained ex vivo with MHC tetramers at days 6 (in blood) and 14 (in splenocytes), and intracellular cytokine IFNγ, IL-2, TNFα secretion of I8V antigen-specific CD8 T cells stained ex vivo with tetramers (day 14; in splenocytes) measurements by flow cytometry (**d**); *n* = 4 and *n* = 5; results presented as mean values +/−SD; one-way ANOVA.

MHC class I and II proteins present antigenic peptides to CD8 and CD4 T lymphocytes, respectively, leading to coordinated T-cell activation^17^. We wanted to assess whether the increased expression of MHC genes upon zabadinostat treatment and increased level of activated T cells translated into engagement with the adaptive immune response. We tested this idea in mice immunised with an immunogenic peptide, called I8V, which is presented by MHC class I proteins and in previous studies has been shown to activate antigen-specific CD8 T lymphocytes^18^. Under conditions of peptide immunisation and treatment with zabadinostat (where there was no apparent effect on body weight; Fig. 4a, b), we monitored the level of the total and antigen-specific CD8 T cells, using the I8V peptide bound to MHC class I tetramers (H2-Kb) to capture antigen-specific CD8 T cells. When the total effector memory CD8 T-cell population was measured (CD8 + CD44 + CD62L− T cells), there was a general increase in the population dependent on peptide vaccine treatment, with an impact from zabadinostat treatment most evident at day 14 (Fig. 4c and Supplementary Fig. 8). This contrasted with antigen-specific CD8 T cells measured against the I8V-peptide immunogen, where there was a significant increase in activated CD8 T cells upon zabadinostat treatment, apparent at day 6 and continuing to day 14 (Fig. 4c and Supplementary Fig. 8). We conclude that zabadinostat enhances antigen-specific CD8 T cells.

We progressed on to characterise the properties of the antigen-specific CD8 T-cell population by measuring interferon IFNγ, IL-2 and TNFα expression, which represents markers for different stages of CD8 T-cell activation^19^. The major difference was seen in the high-level IFNγ-positive CD8 T cells upon treatment with zabadinostat contrasting with IL-2 and TNFα (Fig. 4d and Supplementary Fig. 8). Interferon γ is generally regarded as a marker for activated cytotoxic CD8 T cells^19^, which therefore reaffirms the view that zabadinostat treatment not only causes a quantitative increase in antigen-specific CD8 cells, but also enhances the active cytotoxic population of T cells.

### Zabadinostat enhances the level of CD4 T cells

**Fig. 5:**
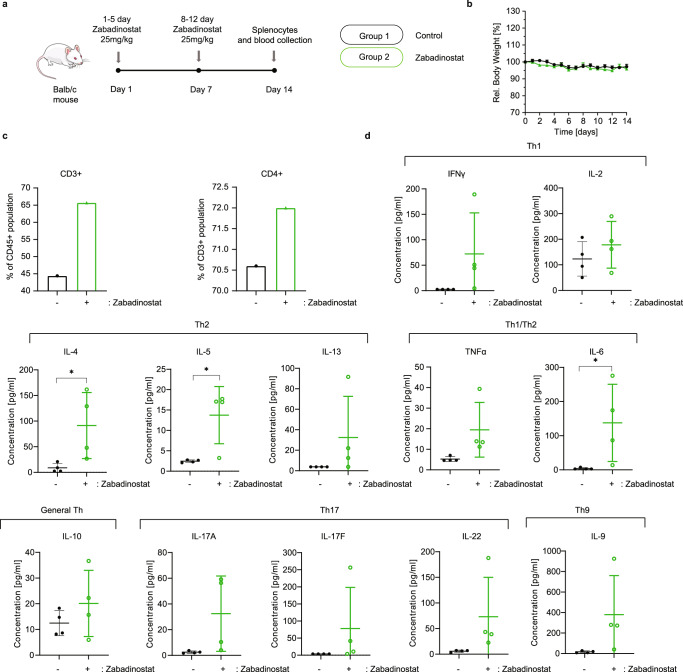
Zabadinostat enhances the CD4 T-cell response. **a** Schematic representation of the experiment with zabadinostat. Balb/c mice were treated with orally administrated zabadinostat at 25 mg/kg for 14 days (5 days on/2 days off) or vehicle only; *n* = 4 per group; **b** relative body weight representation of treated and non-treated mice; *n* = 4; results presented as mean values +/−SD. **c** General T-cell activation and CD4-positive T cells measured by flow cytometry; measurements were performed in pooled splenocytes from four mice; also, we noticed similar percentage of CD8-positive T cells in control and zabadinostat-treated groups (26.2% and 24.3%). The viability of the splenocytes was similar in zabadinostat treatment compare to untreated control (70.9% and 76.6% viable cells, respectively). **d** Analysis of panel of Th1, Th2, Th1/Th2, General Th, Th17 and Th9 cytokines in serum collected from mice treated with zabadinostat and the control group; *n* = 4; results presented as mean values +/−SD; Student’s *t* test.

Given the effect of zabadinostat on activated CD4 T cells (Fig. 3d), we were interested to clarify the nature of the T-cell subsets affected by the treatment. We addressed this question in splenocytes taken from Balb/c mice (Fig. 5a, b) where we found a significant increase in CD45/CD3 and CD3/CD4 T cells upon zabadinostat treatment (Fig. 5c and Supplementary Fig. 9). To substantiate this result and further delineate which T helper (Th) subsets were affected, we measured the level of Th cytokines in the serum (Fig. 5d and Supplementary Figs. 10 and 11). An increase in the level of IL-10 (indicative of the general Th population) was observed, together with increased cytokine levels which represent predominant Th subsets, namely Th1 (IFNγ and IL-2), Th2 (IL-4, IL-5 and IL-13), Th1/Th2 (TNFα and IL-6), Th17 (IL-17A, IL-17F and IL-22) and Th9 (IL-9), further corroborating the effect of zabadinostat on CD4 T helper subsets. However, it was noteworthy that in tissue taken from mice treated with zabadinostat, although there was increased histone acetylation (apparent, for example, in the lung, liver, and colon tissue by immunohistochemistry), there was no apparent quantitative difference in the level of CD8 and CD4 T cells in the tissues examined (Supplementary Figs. 12–14). We conclude therefore that zabadinostat treatment is likely to increase the activated population of CD4 T cells in splenocytes, including the prevalent Th subsets, with limited effect in tissues.

### Zabadinostat augments the immune response against the covid19 spike protein

**Fig. 6:**
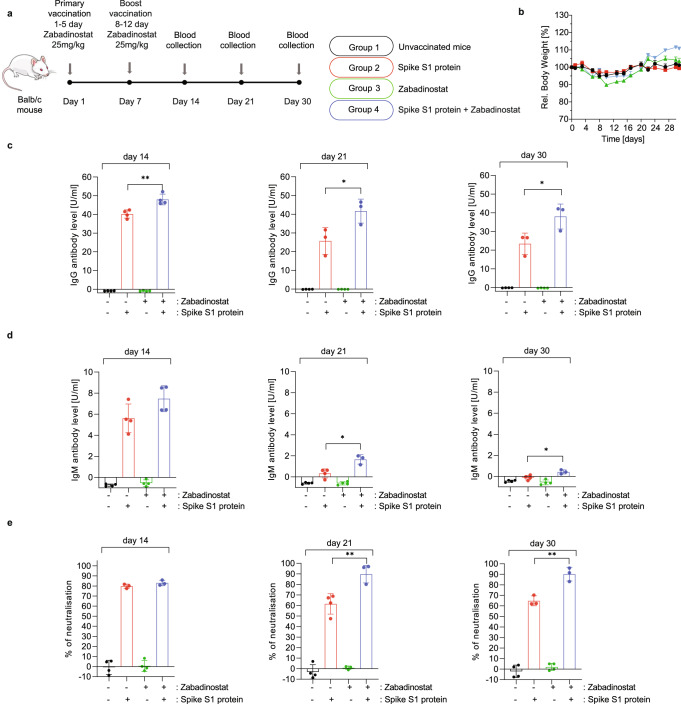
Zabadinostat enhances the antibody response against the covid19 spike S1 protein. **a** Schematic representation of the immunogenicity experiment with zabadinostat and spike S1 protein. Balb/c mice were treated with orally administrated zabadinostat at 25 mg/kg for 14 days (5 days on/2 days off) and intravenous spike S1 protein (days 1 and 7) with respect to vehicle-only control. Blood samples were collected on days 14, 21, and 30; *n* = 4 per group; **b** Relative body weight representation of treated and non-treated mice; n = 4; results presented as mean values +/−SD; Analysis of IgG (**c**) and IgM (**d**) spike protein antibodies at days 14, 21, and 30 in serum collected from mice treated with spike protein, zabadinostat, the combination and the control group; *n* = 4; results presented as mean values +/−SD; one-way ANOVA. **e** Analysis of spike S1-ACE2 neutralising antibodies at days 14, 21, and 30 in serum collected from mice treated with spike protein, zabadinostat, the combination, and the control group; *n* = 4; results presented as mean values +/−SD; one-way ANOVA.

In continuing the analysis, we evaluated the impact of zabadinostat on the response to the covid19 spike protein and investigated the cellular basis of the immune response. Healthy mice were immunised under conditions where there was minimal effect on body weight (Fig. 6a, b) with recombinant purified covid19 spike S1 protein with or without co-treatment with zabadinostat, and antigen-specific IgM and IgG measured. The level of both classes of antibody was higher in the presence of zabadinostat, particularly for IgG where there was a sustained and significant increase over time, up to day 30 post immunisation (Fig. 6c). The effect of zabadinostat on the IgM level again was clear but less marked and was particularly apparent at day 21 where in the absence of zabadinostat treatment IgM levels were not detectable (Fig. 6d). We similarly explored the impact of zabadinostat on neutralising antibodies that disrupt spike S1 protein binding to its cellular receptor ACE2, which in vaccinated humans correlates with protection against viral infection^20^. Remarkably, zabadinostat elevated both the level of neutralising antibody and the durability of the response, which was maintained at a higher level over a longer period of time compared to mice immunised with the spike protein alone (e.g., day 30; Fig. 6e). These results show that for a complex multiple epitope vaccine antigen, like covid19 spike S1 protein, zabadinostat augments the humoral response both quantitatively and temporally.

It was of further interest to explore the level of T cells in covid19 spike S1-immunised mice (Supplementary Fig. 15a–c). Zabadinostat enhanced the T-cell arm (CD45 + CD3+) compared to the negligible effect with the spike S1 protein alone, and there was an increase when the spike S1 protein and zabadinostat were co-administered (Supplementary Fig. 15c). This effect was not only apparent on the CD3/CD4+ T-cell population (Supplementary Fig. 15c), but also seen when the IFNγ and TNFα CD4+ Th cell subsets were measured (Supplementary Fig. 15c).

**Fig. 7:**
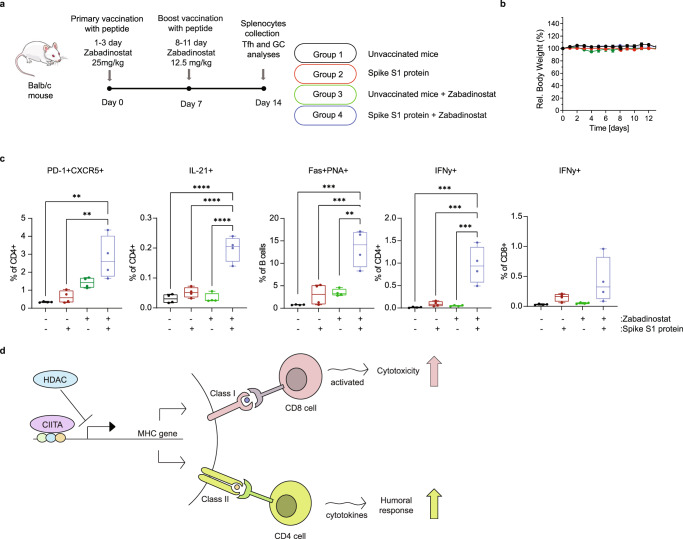
Zabadinostat augments the level of GC B cells through modulating the activity of Tfh cells. **a** Schematic representation of the immunogenicity experiment with zabadinostat and spike S1 protein. Balb/c mice were treated with orally administrated zabadinostat at 25 mg/kg (days 1–3) or 12.5 mg/kg (days 8–11), and intravenous spike S1 protein (10 µg) (days 0 and 7) with respect to vehicle-only control; splenocytes were collected at day 14; **b** relative body weight representation of treated and non-treated mice; *n* = 4; results presented as mean values +/−SD; **c** follicular T-cell level (CD4 + PD-1 + CXCR5 + positive T cells), germinal centre B cells (Fas+ PNA+ positive B cells) and intracellular cytokine IL-21 (in CD4-positive T cells), IFNy (in CD4 and CD8-positive T cells) secretion measurements by flow cytometry; *n* = 4; results presented as mean values +/−SD; one-way ANOVA. **d** Model describing the effect of zabadinostat in regulating the adaptive immune response. It is proposed that inhibition of histone deacetylase induces both cellular and humoral immune responses by increasing antigen presentation (e.g., by dendritic cells), activating T and B cells and increasing germinal centre B cells (stimulated by follicular T cells), which coincides with elevated the MHC class I and II proteins.

We also studied the effect of zabadinostat on the interaction between T-follicular helper cells (Tfh) and germinal centre (GC) B cells. Tfh cells are a specialised subset of CD4+ T cells that play a critical role in protective immunity by helping B cells in germinal centres to undergo rapid proliferation and produce antibody against foreign pathogens^21^. We explored whether Tfh and GC B cells were affected by zabadinostat treatment in response to the spike protein in immunised mice. To this end, mice were treated with zabadinostat as described (Fig. 7a, b), and splenocytes were harvested and analysed for Tfh and GC B cells. For Tfh CD4 cells, we measured the level of the chemokine receptor 5 (CXCR5), programmed cell death-1 (PD-1) and the cytokine IL-21, and for GC B cells we measured Fas and PNA^22,23^. We observed a significant increase in Tfh cells in spike immunised mice upon zabadinostat treatment, and the same was apparent when the GC B cells were measured (Fig. 7c and Supplementary Fig. 16). In the same treatment conditions, both CD4 and CD8 T cells exhibited increased activity reflected in the higher levels of IFNy (Fig. 7c and Supplementary Fig. 16). These results highlight the ability of zabadinostat to augment the level of GC B cells through modulating the activity of Tfh cells, and more generally the activity of CD4 and CD8 cells.

## Discussion

As a class of drugs, HDAC inhibitors have found clinical utility in cancer^6^, reflecting the widespread deregulation of lysine acetylation processes during tumourigenesis^4^. It is however clear that despite intense efforts, relatively few HDAC inhibitors have been approved for clinical use. For the most part, approvals have been seen in haematological malignancies such as peripheral and cutaneous T-cell lymphoma and multiple myeloma^24–26^. To our knowledge, none of them has been described to possess the properties of a stand-alone immune-modulating agent in healthy cells and animals, although some studies have documented the effect of HDAC inhibitors on the cancer immune response^27–29^. Our study, which describes the systemic effect of zabadinostat on the adaptive immune response in healthy animals, therefore represents a highly significant advance in the field.

In previous work, we suggested that the increased MHC gene expression in tumour cells was the basis of the synergistic anti-tumour activity seen when zabadinostat is combined with immune checkpoint inhibitors^10^, reflecting enhanced CD4 and CD8 T lymphocytes in the tumour micro-environment^10^. We reasoned therefore that the enhanced T-cell infiltration in the tumour reflected not only the release of T cells from a PD-1-PDL1 checkpoint blockade, but also engagement of T cells with the increased MHC class I and class II expression. Although of great significance to cancer therapy and informative on how best to deploy zabadinostat as a cancer agent, it was of high interest to assess whether zabadinostat had effects on an uncompromised immune system.

The results presented here show that zabadinostat treatment can induce MHC class I and II gene expression and increase protein levels in normal cells and healthy tissue and activate a variety of cell types involved in the adaptive immune response. The increased MHC expression occurred in cells grown in vitro and importantly in tissue from mice dosed with zabadinostat. At a mechanistic level, we identified *CIITA* as a gene required for zabadinostat to increase MHC gene expression in normal cells. *CIITA* is a transcriptional regulator involved in the control of MHC gene expression, which some reports suggest is a lysine acetyl-transferase that preferentially targets MHC class II genes^30^. Interestingly, *CIITA* assisted the acetylation of chromatin in the promoter region of MHC genes in zabadinostat-treated cells, thus providing a possible explanation for its positive impact on MHC gene transcription (Fig. 7d).

Because CD8 and CD4 T lymphocytes engage with MHC class I and II proteins, we evaluated whether zabadinostat affected CD8 and CD4 T cells. Zabadinostat increased the level of activated CD4 and CD8 T lymphocytes, with a similar effect on B cells. With a peptide vaccine which stimulates an antigen-specific CD8 T-cell response^18^, zabadinostat resulted in elevated levels of activated antigen-specific CD8 T cells, with a prominent increase in the level of IFNγ positive CD4 and CD8 cells reflecting activated cytotoxic T cells^19^. These results highlight the systemic effect that zabadinostat has on activating the adaptive T-cell response.

We also assessed the effect of zabadinostat on DCs, and on the Tfh-GC B-cell interaction. There was a greater level of mature activated DCs upon treatment, including increased MHC class II expression, suggesting that the ability of DCs to present antigen to, for example, B cells was enhanced upon zabadinostat treatment. This idea was tested by measuring the antibody response against covid19 S1 spike protein in a mouse study, where there was a significant increase in antibody level (IgM and IgG) in treated mice compared to mice vaccinated with the spike S1 alone, an effect that was also of increased durability compared to the spike alone treatment. There were more activated CD4 and CD8 T lymphocytes (reflecting increased IFNγ), and the Tfh-GC B-cell interaction was enhanced upon zabadinostat treatment. This reflected elevated neutralising antibodies that block the binding of spike S1 protein to its ACE2 receptor, which is the major route for covid19 to gain entry into human cells and cause its pathology^31,32^. Potentially, combining zabadinostat with a covid19 spike vaccine will augment the humoral and cell-mediated arms of the immune response, providing a stronger and longer response to vaccination. The effect that zabadinostat has on different levels combine to deliver an efficient immune response; namely enhanced MHC expression, increased antigen presentation by DCs and activated T and B cells, and significantly more GC B cells stimulated by Tfh T cells.

Overall, our results establish zabadinostat as a systemic regulator of the adaptive immune response, and its capacity to augment both the cellular and humoral arms of the response (Fig. 7d). This remarkable property was observed in otherwise healthy animals and therefore argues that zabadinostat can be deployed where there could be a clinical benefit derived from enhancing the immune response, potential scenarios being the vaccination of individuals against either infectious disease or cancer.

## Methods

### Cell culture and compound treatment

Human colorectal adenocarcinoma HCT116 (ATCC® CCL-247; RRID:CVCL_0291),two normal human bronchial epithelium BEAS2B (ATCC® CRL-9609; RRID:CVCL_0168) and NBE1 (RRID:CVCL_9Y83), were cultured in Dulbecco’s modified Eagle medium (DMEM) (D6429, Sigma-Aldrich, St. Louis, MO, USA) supplemented with 10% foetal bovine serum (FCS-SA, Labtech, Heathfield, UK) and 1% penicillin/streptomycin (15140-122, Gibco, Life Technologies, Carlsbad, CA, USA). Jurkat (donated by Prof. Eric O’Neill, Department of Oncology, University of Oxford), CEM, and Raji cell lines (donated by Prof. Quentin Sattentau, Sir William Dunn School of Pathology, University of Oxford), were cultured in RPMI-1640 Medium (R8758, Sigma-Aldrich, St. Louis, MO, USA) supplemented with 10% foetal bovine serum (FCS-SA, Labtech, Heathfield, UK) and 1% penicillin/streptomycin (15140-122, Gibco, Life Technologies, Carlsbad, CA, USA). All cell lines were tested for mycoplasma contamination before use. Zabadinostat/CXD101 was dissolved in DMSO and used as described^10^.

### siRNA treatment

RNA interference was performed with 50 nM siRNA for 2 days using the Oligofectamine transfection reagent (12252011, Invitrogen, Carlsbad, CA, USA), as per the manufacturer’s instructions. Sequences for siRNA are as follows: nontargeting control, 5′-AGCUGACCCUGAAGUUCUU-3; CIITA (human), 5’-UCUCCAGUAUAUUCAUCUA-3’.

### RNA isolation and quantitative RT-PCR

RNA was isolated from cells using TRIzol (15596026, Invitrogen) or the Direct-zol RNA MiniPrep kit (R2072, Zymo Research, Irvine, CA, USA) according to the manufacturer’s instructions. 1 µg of total RNA was used for complementary DNA (cDNA) synthesis. Reverse transcription with oligo(dT)20 primer (N8080128, Invitrogen) was performed using SuperScript III Reverse Transcriptase (18080-044, Invitrogen) as per the manufacturer’s instructions. Quantitative reverse transcription PCR (qRT-PCR) was carried out in technical triplicate using the indicated primer pairs and the Brilliant III Ultra-Fast SYBR® Green QPCR Master Mix (600882, Agilent, Santa Clara, CA, USA) on an AriaMX real-time qPCR instrument (AriaMX, Agilent). Primers for human MHC genes: *HLA-A*, F: 5’-GATCACCAAGCGCAAGTG-3’, R: 5’-GGTGGTGGGTCATATGTGTC-3’; *HLA-B*, F: 5’-GCCGCGAGTCCGAGAGA-3’, R: 5’-GCCTTGTAGATCTGTGTGTTCC-3’; *HLA-C*, F: 5’-GGAGACACAGAAGTACAAGCG-3’, R: 5’-CGTCGTAGGCGTACTGGTCATA-3’; *HLA-E*, F: 5’-TCATCTCTGTGGGCTACGTG-3’, R: 5’-TCAGACCCCCAGAATCTCAC-3’; *HLA-DMB*, F: 5’-CACTTACACCTGTGTGGTAGAGC-3’, R: 5’-GCAGACACAGAAACCTTCAGG-3’; *HLA-DOA*, F: 5’-ATCGCCGCAATCAAAGCCCATC-3’, R: 5’-TGCAGATGAGGATGTTGGGCTG-3’; *HLA-DOB*, F: 5’-CCAGATGCTGAGCAGTGGAACA-3’, R: 5’-GGTACACTGTCACCTCTGGTTG-3’; *HLA-DPB1*, F: 5’-GTGCAGACACAACTACGAGCTG-3’, R: 5’-CCTGGGTAGAAATCCGTCACGT-3’; *HLA-DRA*, F: 5’-GATCACCAATGTACCTCCAGAG-3’, R: 5’-GTCTCTGACACTCCTGTGGTG-3’; *HLA-DRB1*, F: 5’-AGCGGCGAGTCCAACCTAAG-3’, R: 5’-CATCCCAGCCTTCTCTTCCTG-3’. Primers for mouse MHC genes: *H2-D1*, F: 5’-TCCTCCGTCCACTGACTCTT-3’, R: 5’-CACAAAAGCCACCACAGCTC-3’; *H2-K1*, F: 5’-GGCAATGAGCAGAGTTTCCGAG-3’, R: 5’-CCACTTCACAGCCAGAGATCAC-3’; *H2-M3*, F: 5’-CATGGTGGTGGTCTCTTTG-3’, R: 5’-GAATGTGAGCCAGAACCTG-3’; *H2-Q1*, F: 5’-GGAGCAGAATTACACATGCC-3’, R: 5’-AGGAGGCTCCCATCTCAG-3’; *H2-Q4*, F: 5’-GGAGCAGAATTACACATGCC-3’, R: 5’-GAGGAGGCTCCCATCTCA-3’; *H2-T3*, F: 5’-GGCAGCAGAGATCACCAGAA-3’, R: 5’-AATCCTTGCATGGGCCCTC-3’; *H2-Aa*, F: 5’-TCTGACGATGACATTTATGACTG-3’, R: 5’-ATCTCAGGTTCCCAGTGTTTC-3’; *H2-DMA*, F: 5’-ATTCACACTGAAGCCCCTGG-3’, R: 5’-GTCAGGGTCGGTGGAAAGAG-3’; H2-*DMB1*, F: 5’-CCTTCCCACAGCAACAAGGA-3’, R: 5’-AGGAAGGGGTTAGGGCTAGG-3’; *H2-DMB2*, F: 5’-TCATCATCTTCTGTGTTGGC-3’, R: 5’-GGAGTGTAGCTGGAGGAATG-3’, *H2-EB1*, F: 5’-ACGGTGACTGTGTACCCCA-3’, R: 5’-TGACTTCAATGTTGCCAGGGT-3’; *H2-EB2*, F: 5’-TGCCTCAGTAGACAGGTGCAGA-3’, R: 5’-AGAGCAGACCAGGAGGTTATGG-3’. Results were expressed as average (mean) fold change compared to control treatments using the ΔΔCt method from three biological repeat experiments. Glyceraldehyde-phosphate dehydrogenase (*GAPDH*) primer sets were used as an internal calibrator. Error bars represent SE unless otherwise indicated.

### Chromatin immunoprecipitation (ChIP)

BEAS2B cells were transfected with control siRNA or siCIITA for 2 days and treated with DMSO or 1uM zabadinostat for 3 days. ChIP samples were prepared as described previously^33^. Immunoprecipitations were performed using 3 μg of appropriate antibody (control rabbit IgG, anti-Histone H3 [ab1791, Abcam], anti-Histone H3 acetyl K9 [ab10812, Abcam]) and pre-blocked protein A beads. The recovered DNA was purified, and real-time PCR was performed in triplicate with Brilliant III Ultra-Fast SYBR green QPCR master mix on an AriaMx QPCR instrument (Agilent). DNA occupancy was investigated by calculating the percentage enrichment of input for IgG, H3, H3K9 ChIP from triplicate biological repeat experiments. In all cases, the presented figure displays SD unless otherwise stated. ChIP primers for human MHC genes: *HLA-A*, F: 5’-CCCAGTTCTCACTCCCATTG-3’, R: 5’-GTAGCAGGAGGAGGGTTCG-3’; *HLA-B*, F: 5’-CTTCCAGGATACTCGTGACGC-3’, R: 5’-CCATGACCAGCATCTCGG-3’; *HLA-C*, F: 5’-CAATTCCCACTCCCATTGG-3’, R: 5’-CGAGAGCAGCAGGAGGAG-3’; *HLA-E*, F: 5’-CTCTCGTAACCTGGTCATGTGTC-3’, R: 5’-CATCTACCATGATCCCAGCC-3’; *HLA-DMB*, F: 5’-CTTTTGGGAGAGCATTGAGAAG-3’, R: 5’-GGTAAACGTCATCCTGCCTTAG-3’; *HLA-DOA*, F: 5’-CAGCAACAGATACATTCACTCAGAG-3’, R: 5’-GGGCCATTACACTCTGGTG-3’; *HLA-DOB*, F: 5’-GTGAAAGTATCCTACCCAGTACCTG-3’, R: 5’-CAGCCTCTTCAGAATGAGCTC-3’; *HLA-DPB1*, F: 5’-CCCAAACATCATGACTTATCTGAC-3’, R: 5’-CCCTGGGATTGGACAGAG-3’; *HLA-DRA*, F: 5’-GAGTATCTTGTGTCCTGGACCC-3’, R: 5’-GGGAGTGAGGCAGAACAGAC-3’; *HLA-DRB1*, F: 5’-CATGCAACTGGTTCAAACC-3’, R: 5’-CTGATTTCCTTGCTCCTGG-3’.

### Western blotting

Cell pellets were lysed in radioimmunoprecipitation assay buffer (50 mM tris-HCl (pH 8), 150 mM NaCl, 1% Igepal CA-630, 0.5% sodium deoxycholate, 0.1% SDS, 0.2 mM sodium orthovanadate, and protease inhibitor cocktails), for 30 min on ice and centrifuged for another 30 min at maximum speed at 4 °C. Protein concentration was assessed by Bradford assay (B6916, Quick Start™ Bradford 1× Dye Reagent, Bio-Rad Laboratories, Hercules, CA, USA). After gel electrophoresis, proteins were transferred onto the PVDF or nitrocellulose membrane by means of Trans-Blot® Turbo™ Transfer System (1704150, Bio-Rad Laboratories) and blocked by 1-h incubation in 5% skimmed milk (70166, Merck Group, Darmstadt, Germany) in PBST at room temperature. The following antibodies were used in immunoblotting: anti-Histone H3 (ab1791, Abcam; 1:1000), anti-H3AcK9 (ab10812, Abcam; 1:1000), anti-H3AcK14 (#7627, Cell Signaling, Danvers, MA, USA; 1:1000), anti-β-Actin (#3700, Cell Signaling; 1:1000), anti-CIITA (TA319682, Origene, Rockville, MD, USA; 1:1000) all overnight at 4 °C. Next, membranes were washed and treated with secondary antibody for 1 h at RT. Chemiluminescent signals were detected by LICOR C-Digit (LI-COR Biosciences, Lincoln, NE, USA).

### MTT assay

Cells were seeded onto 96-well plates overnight and the next day dosed with zabadinostat and incubated for 3 days. Next, 10 µl of 12 mM Thiazolyl Blue Tetrazolium Bromide (M-2128, MTT, Sigma-Aldrich, MO, USA) was added into a well (final concentration 5 µM) and incubated for 2 h at 37 °C. After the labelling, the medium was discarded and formazan crystals were dissolved in 50 µl of DMSO (D2650, Sigma-Aldrich) by shaking for 15 min. Absorbance was read by TECAN sunrise plate reader (Tecan, Switzerland) at the 584 nm wavelength. Data were analysed, and IC_50_ doses were calculated in GraphPad Prism 8 (GraphPad Software, USA).

### Immunogenicity peptide experiment

Twenty C57BL/6 female mice (5 mice per group: control; zabadinostat-treated; peptide vaccinated; zabadinostat/peptide vaccinated) at age 26 weeks were purchased from Charles River Discovery Research Services (Germany). Ten mice were vaccinated intravenously with 30 µg of peptide encoding CD8 T-cell epitopes from the beta-galactosidase gene 497–504 (ICPMYARV; I8V, H2-Kb) (Genscript) with 30 µg polyIC (vac-pic, InVivogen) and 15 µg anti-CD40 (anti-CD40, 2bScientific). One day post vaccination, half the mice were administered zabadinostat (25 mg/kg) by oral gavage once a day for 5 days. Mice were boosted at day 7 with peptides in the presence of polyIC and anti-CD40 as before. One day post boost, the mice were again treated with zabadinostat (25 mg/kg) once a day for 5 days. Mice were bled at day 6 post prime and the levels of peptide-specific CD8 T cells were measured by ex vivo staining with MHC tetramers (produced by the NIH Tetramer Core Facility, Emory University, USA; incubated 20 min at 37 °C) followed by flow cytometry using BD LSRII flow cytometer (Oxford, UK), as previously described^18,34^. Mice were culled at day 14 post prime, and the splenocytes were restimulated with the autologous peptide in the presence of brefeldin A for 18 h. The levels of epitope-specific CD8 T cells measured by ex vivo staining with MHC tetramers and intracellular cytokines IFNγ, IL-2, TNFα secretion of I8V epitope-specific CD8 T cells stained ex vivo with tetramers was measured by flow cytometry.

### Immunogenicity zabadinostat experiment

All experiments and protocols were approved by the animal welfare body at Charles River Discovery Research Services Germany (where each experiment was performed) and the local authorities, and were conducted according to all applicable international, national and local laws and guidelines. Four mice in each group were administered zabadinostat (25 mg/kg) or vehicle by oral gavage once a day for 5 days and then again at days 8–12. On day 14, spleens of study mice were harvested, pooled group-wise and splenocytes suspensions was prepared. Pooled splenocytes were stained for the flow cytometry panel: CD3 (100210, Biolegend, San Diego, CA, USA; 1:100), CD45 (103132, Biolegend; 1:100), and CD4 (558107, BD Biosciences, New York, USA; 1:100), IFNγ (554413, BD Biosciences; 1:100), TNFα (12-7321-82, eBiosciences, Waltham, MA, USA; 1:100), and live/dead marker (423102, Biolegend). Data were acquired on an Attune NXT flow cytometer (ThermoFisher, Waltham, MA, USA) and results were analysed using FlowJo Software. From this experiment, serum was collected from each mouse, and the panel of twelve cytokines (IFNγ, IL-2, IL-4, IL-5, IL-13, IL-10, IL-9, TNFα, IL-6, IL-17A, IL-17F, IL-22) was analysed using LEGENDplex MU Th Cytokine Panel (12-plex) VbP V03 according to manufacturer’s instruction (741044, Biolegend).

### Immunogenicity spike protein experiments

All experiments and protocols were approved by the animal welfare body at Charles River Discovery Research Services Germany (where each experiment was performed) and the local authorities and were conducted according to all applicable international, national and local laws and guidelines. In total, 16 mice were vaccinated intravenously with 10 µg of full-length Spike S1 subunit protein (Z03501, Genscript) with 30 µg polyIC (InVivogen) and 15 µg anti-CD40 (2bScientific). One day later, half the mice were administered zabadinostat (25 mg/kg) by oral gavage once a day for 5 days. Mice were boosted at day 7 with spike protein in the presence of polyIC and anti-CD40 as before. One day post boost, the mice were again treated with zabadinostat (25 mg/kg) once a day for 5 days. On day 14, spleens from four mice in each group were harvested, pooled group-wise and splenocyte suspensions were prepared. Pooled splenocytes were stimulated with spike protein (40 µg/ml), PMA/Ionomycin solution or remained unstimulated. After incubation, cells were stained for the flow cytometry panel: CD3 (clone REA606, Biolegend, 1:100), CD45 (clone 30-F11, Biolegend, 1:100), CD4 (clone RM4-5, BD Biosciences, 1:100), CD8 (clone 53-6.7, Biolegend, 1:100), IFNγ (clone XMG1.2, BD Biosciences, 1:100), TNFα (clone MP6-XT22, eBiosciences, 1:100), and live/dead marker (Biolegend). Data were acquired on an Attune NXT flow cytometer (ThermoFisher) and results were analysed using FlowJo Software. From this experiment, serum from each mouse was collected as well. From the remaining 16 mice, serum was collected at days 14, 21, and day 30 when the mice were culled.

### Spike protein antibody experiment

The level of IgM and IgG spike-specific antibodies was analysed in serum collected from immunised mice at days 14, 21 and 30. The analysis was performed using mouse anti-SARS-CoV-2 (COVID) spike protein 1 (S1) IgG (RV-405220) and IgM (RV-405230) kits according to the manufacturer’s protocol (Alpha Diagnostic, San Antonio, TX, USA). Briefly, the samples were diluted 1000 times with Working Sample/Conjugate Diluent and Low NSB Sample Diluent. Then, samples, calibrators (standards), and positive and negative controls were added to the predetermined wells and incubated for 60 min at room temperature. After washing, cells were incubated for 30 min with anti-mouse IgG or IgM HRP antibody and washed again. Next, TMB substrate was added to each well, and after 15 min of incubation, “stop” solution was added. Absorbance was read at 450 nm and 650 nm as a background, using TECAN sunrise plate reader. Values were calculated from the standard curve.

### Spike neutralisation antibody assay

The level of neutralising antibodies was analysed in serum collected at days 14, 21 and 30 during the above-mentioned experiments. The analysis was performed using SARS-CoV-2 Surrogate Virus Neutralisation Test (sVNT) Kit (RUO) (L00847-A, Genscript) according to the manufacturer’s protocol. Briefly, serum samples were incubated with the diluted RBD domain conjugated with horseradish peroxidase (HRP) solution at 37 °C for 30 min. Next, samples were moved to the 96-well ACE2 protein-coated plate and incubated for 15 min at 37 °C. After being washed four times, to each well TMB (tetramethylbenzidine) solution was added and the plate was incubated in the dark at 20–25 °C for 15 min. To quench the reaction, Stop solution was added and absorbance was read at 450 nm and 650 nm as a background, using TECAN sunrise plate reader.

### Bone marrow-derived dendritic cells

Five 6–8 weeks female Balb/c (Charles River Laboratories) and C57BL/6 mice (donated by Biomedical Services, University of Oxford) were humanely sacrificed by CO_2_ (animals were housed in specific pathogen-free conditions at the Biomedical Services Building, University of Oxford). All work was performed under UK Home Office license PPL PP3430109 in accordance with the UK Animal (Scientific Procedures) Act 1986. All work was performed by trained and licensed individuals, and two femurs and tibias of the mice were prepared to harvest bone marrow cells. The bone marrow cells were isolated by flushing the bone cavity with a sterile cold RPMI medium. The bone marrow cells were resuspended in 1 ml ACK lysis buffer (Lonza) for 2 min to lyse the red blood cells, then stopped with 20 ml 10% FBS/RPMI medium, followed by centrifugation at 1500 rpm, 5 min at room temperature. Next, cells were seeded, treated with GM-CSF (250 IU/ml) and IL-4 (5 IU/ml) containing medium, and incubated at 37 °C in 5% CO_2_. Medium was half changed on day 3. At day 5, cells were treated with GM-CSF (Z03300-50, Genscript), IL-4 (Z02996-50, Genscript) and LPS (L3129-10mg, Sigma) to mature dendritic cells. After incubation for 24 h, DC cells were treated with 1 µM zabadinostat for another 48 h, harvested, and the phenotype was analysed by FACS. The following antibodies were used: CD11c (557400, BD Pharmingen, 1:100), CD86 (561964, BD Pharmingen 1:100).

### MHC class I and II expression analysis

NBE1, BEAS2B, Jurkat, CEM, and Raji cells were treated with 1 µM abadinostat for 48 h, stained with HLA-ABC (560965, BD Pharmingen, 1:100) and HLA- DR, DP, DQ (562008, BD Pharmingen, 1:100) antibodies. Bone marrow-derived DCs were harvested and stained with CD11c, CD86 and/or H2- I-A/I-E (107613, Biolegend, 1:100) and/or H2 class I (566776, BD Pharmingen, 1:100) antibodies. After 30 min incubation at room temperature, cells were analysed by flow cytometry (BD Accuri C6, Becton Dickinson). Additionally, the level of acetylation and histone H3 was assessed after fixation (4% formaldehyde solution for 15 min, room temperature) and permeabilisation (0.5% Triton X-100 solution for 15 min, room temperature) of the DCs with H3 (Abcam, 1:500), H3AcK14 (Cell Signaling, 1:500) antibodies, AlexaFluor™ 594 (a21207, Life Technologies, 1:1000).

### T-cell and B-cell activation experiment

Five 6–8-weeks female Balb/c (Charles River Laboratories) were humanely sacrificed by CO_2_ (all animals were housed in specific pathogen-free conditions at the Biomedical Services Building (University of Oxford). All work was performed under UK Home Office license PPL PP3430109 in accordance with the UK Animal (Scientific Procedures) Act 1986. All work was performed by trained and licensed individuals), their spleens were removed, and passed through a 40-µm cell strainer (Falcon), and the single-cell suspension was pelleted by centrifugation. The splenocytes were resuspended in 1 ml ACK lysis buffer (Lonza) for 3–5 min to lyse the red blood cells, then stopped with 20 ml PBS, followed by centrifugation at 1500 rpm, 5 min at room temperature. The splenocyte pellet was resuspended in RPMI medium, splenocytes were seeded and treated with 1 µM zabadinostat or PHA-L (11249738001, Merck) for 48 h. Activation of CD8, CD4, and B cells was evaluated by flow cytometry (BD Accuri C6). The following antibodies were used: CD3 (561826, BD Pharmingen, 1:100), CD8 (553032, BD Pharmingen, 1:100), CD4 (557308, BD Pharmingen, 1:100), CD20 (150409, Biolegend, 1:100), CD69 (557392, BD Pharmingen, 1:100).

### Immunohistochemistry on tissue

Lung, liver, and colon tissues were harvested at day 14, fixed, and embedded in paraffin blocks and cut into 5-µm sections. FFPE slides were washed for 5 min with Histochoice (H2779, Sigma-Aldrich), followed by two times of 3-min washing in 100% ethanol, 3 min in 70% ethanol and 5 min in tap water. Next, samples were incubated with antigen retrieval solution (Tris/EDTA [443866G, VWR, PA, USA and 6381-92-6, MP Biomedicals, Belgium, respectively]) at 99 °C in water bath for 20 min. After 3× washing with purified water, samples were incubated in freshly made 6% methanol/H_2_O_2_ for 15 min and washed in tap water. In the next steps, slides were washed in 1% PBST for 5 min, blocked in blocking serum solution (PK-4001, Vectastatin ABC kit, Vector Laboratories, Burlingame, CA, USA) for 20 min, washed again in 1% PBST for 5 min and incubated overnight at 4 °C with primary antibodies: anti-H3AcK9 (ab10812, Abcam; RRID:AB_297491, 1:3000), anti-CD4 (ab183685, Abcam, 1:3000), and anti-CD8 (ab203035; Abcam, 1:3000). Samples were further stained with secondary antibody (Vectastain ABC kit) at room temperature. In the next step, ABC solution (PK-4001, VECTASTAIN® ABC-HRP Kit, Peroxidase, Rabbit IgG, Vector Laboratories) was added for 30 min, slides were washed in 1% PBST and incubated with DAB solution (SK-4100, Vector Laboratories) for another 10 min. Sections were counterstained with hematoxylin (MHS16, Sigma-Aldrich). Results were analysed using Leica DM2500 optical microscope and presented as semi-quantitative using ImageJ software (National Institutes of Health, USA).

### GC B-cell and Tfh cell phenotyping experiment

Sixteen 8-weeks-old female Balb/c mice were vaccinated intravenously with 10 µg of full-length Spike S1 subunit protein (Z03501, Genscript) with 30 µg polyIC (InVivogen) and 15 µg anti-CD40 (2bScientific) or vehicle only. One day post vaccination, half the mice were administered zabadinostat (25 mg/kg) by oral gavage once a day for 3 days. Mice were boosted at day 7 with spike protein in the presence of polyIC and anti-CD40 as before. One day post boost, the mice were again treated with zabadinostat (12.5 mg/kg) once a day for 4 days. All animals were housed in specific pathogen-free conditions at the Biomedical Services Building (University of Oxford). All work was performed under UK Home Office license PPL PP3430109 in accordance with the UK Animal (Scientific Procedures) Act 1986. All work was performed by trained and licensed individuals.

### Splenocyte dissociation

Splenoctyes were dissociated as previously described^2^. Spleens were crushed through a 70-micron strainer, which was flushed as needed with R5 media (RPMI-1640 + 5% FBS + 1% penicillin/streptomycin). Cells were pelleted at 1500 rpm for 5 min and the supernatant was discarded. Red blood cells were lysed using 1× BioLegend Red Blood Cell lysis buffer for 3 min. Lysis buffer was neutralised by the addition of R5, cells were pelleted at 1500 rpm for 5 min, and the supernatant was discarded. One additional round of washing with R5 media was performed before cells were resuspended at 2 × 10^7^ cells per ml in R10 media (RPMI-1640 + 10% FBS + 1% penicillin/streptomycin).

### T_FH_ cell phenotyping

Identification of CXCR5^+^PD-1^+^ T_FH_ cells was performed as previously described^23^, with modification. Splenocytes (200 μl) were plated in a U-bottom 96-well plate and washed one time with FACS buffer (PBS + 1 mM EDTA + 0.05% BSA). Cells were then stained for 30 min at 4 °C with biotinylated anti-CXCR5 antibody (clone L138D7, 1:100 dilution, BioLegend). Cells were washed two times with FACS buffer. The remaining staining cocktail was then added: streptavidin PE-Cy7 (1:1000), anti-PD-1 APC (clone RMP1-30, 1:50), anti-CD8 AlexaFluor 700 (clone 53–6.7, 1:100), anti-B220 APC-Cy7 (clone RA3-6B2, 1:100), anti-F4/80 APC-Cy7 (clone BM8, 1:100), anti-CD4 Brilliant Violet 650 (clone RM4-5, 1:100), anti-CD44 Brilliant Violet 785 (clone IM7, 1:100), and Near-IR Live/Dead dye (1:400). All reagents from BioLegend except viability dye which was from ThermoFisher. After 30 min at 4 °C, cells were washed two times with FACS buffer. Cells were fixed for 20 min at 4 °C using the Cytofix/Cytoperm kit (BD Biosciences). Following two final washes in FACS buffer, samples were stored at 4 °C until data acquisition on a BD Fortessa flow cytometer. Data analysis was performed using FlowJo v. 10.8.1.

### Intracellular cytokine staining

Intracellular cytokine staining was performed as previously described^22^. Briefly, splenocytes (100 μl) were plated in a U-bottom 96-well plate. Cells were stimulated with 1 μg/ml of overlapping SARS-CoV-2 spike peptide pool covering the entire protein (15 mers overlapping by 11; JPT peptides). Brefeldin A (1:1000, BioLegend) was added at the time of stimulation and cells were cultured at 37 °C, 5% CO_2_ for 5 h. After stimulation, cells were washed one time with FACS buffer (PBS + 1 mM EDTA + 0.05% BSA). Cells were stained for 10 min at 4 °C with Near-IR Live/Dead dye (1:400, ThermoFisher). Cells were washed twice with FACS buffer. Cells were fixed for 20 min at 4 °C using the Cytofix/Cytoperm kit (BD Biosciences), followed by two washes with 1× Perm/Wash buffer (BD Biosciences). Staining for IL-21 was performed using a mouse IL-21R-Fc fusion protein (1:50, R&D Systems) for 30 min at 4 °C. After two washes with 1× Perm/Wash buffer, cells were stained with anti-human Fc-PE (AB_2337681, 1:50, Jackson ImmunoResearch) for 30 min at 4 °C. Cells were washed two times with 1× Perm/Wash buffer. The final round of staining was for 30 min at 4 °C comprised a cocktail of: anti-IFNγ APC (clone XMG1.2, 1:100), anti-CD8 AlexaFluor 700 (clone 53-6.7, 1:100), anti-CD4 Brilliant Violet 650 (clone RM4-5, 1:100), and anti-CD44 Brilliant Violet 785 (clone IM7, 1:100). All antibodies from BioLegend. Two final washes with 1× Perm/Wash buffer were performed before storage of the cells at 4 °C in FACS buffer for acquisition on a BD Fortessa flow cytometer. Data analysis was performed using FlowJo v. 10.8.1.

### GC B-cell phenotyping

Identification of Fas^+^PNA^+^ GC B cells was performed as previously described^22^, with slight modification. Splenocytes (200 μl) were plated in a U-bottom 96-well plate and washed one time with FACS buffer (PBS + 1 mM EDTA + 0.05% BSA). Cells were then stained for 30 min at 4 °C with mouse TruStain FcX (clone 93, 1:50, BioLegend). Cells were washed two times with FACS buffer. The surface cocktail was then added: Peanut agglutinin (PNA) FITC (1:5000, Vector Labs), anti-Fas APC (clone 15A7, 1:50, eBioscience), anti-B220 AlexaFluor 700 (clone RA3-6B2, 1:100, BioLegend), anti-F4/80 APC-Cy7 (clone BM8, 1:100, BioLegend), anti-NK1.1 APC-Cy7 (clone PK136, 1:100, BioLegend), anti-CD3 Brilliant Violet 650 (clone 17A2, 1:100, BioLegend), and Near-IR Live/Dead dye (1:400 dilution, ThermoFisher). After 30 min at 4 °C, cells were washed two times with FACS buffer. Cells were fixed for 20 min at 4 °C using the Cytofix/Cytoperm kit (BD Biosciences). Following two final washes in FACS buffer, samples were stored at 4 °C until data acquisition on a BD Fortessa flow cytometer. Data analysis was performed using FlowJo v. 10.8.1.

### Statistics and reproducibility

Statistical analyses were performed using two-tailed, unpaired Student’s *t* test and a one-way ANOVA test with GraphPad prism 8 Software (GraphPad Software; RRID:SCR_002798). Data are shown as means with SD displayed unless otherwise indicated. *P* values lower than 0.05 were considered significant and are labelled by asterisks (*) for *P* < 0.05, (**) for *P* < 0.01, (***) for *P* < 0.001 and (****) for *P* < 0.0001. All experiments were performed in at least three biological replicates. The exact number of biological replicates is given in every figure legend (i.e., *n* = 3).

### Reporting summary

Further information on research design is available in the Nature Portfolio Reporting Summary linked to this article.

## Supplementary information

Supplementary information

Description of Additional Supplementary Files

Supplementary Data 1

Reporting Summary

## Data Availability

All data generated or analysed during this study (and its supplementary information files) are included in this published article (Supplementary Data 1 for the numerical source data, Supplementary Figs. 17 and 18 for the uncropped immunoblots).
