# Supplementary material for: The HDAC inhibitor zabadinostat is a systemic regulator of adaptive immunity
Source: Commun Biol. 2023 Jan 26;6:102. doi: 10.1038/s42003-023-04485-y (PMC9878486; doi:10.1038/s42003-023-04485-y)
Supplement: Supplementary file 1 — Supplementary information [file 42003_2023_4485_MOESM1_ESM.pdf]

Supplementary information for

The HDAC inhibitor zabadinostat is a systemic regulator of adaptive immunity

Geng Liu[~1], Wojciech Barczak[~1], Lian Ni Lee[2], Amit Shrestha[1], Nicholas M Provine[3] Gulsah Albayrak[1], Hong Zhu[1,4], Claire Hutchings[2], Paul Klenerman[2,3] and Nicholas B La Thangue[*1,5]

[1] Laboratory of Cancer Biology, Department of Oncology, University of Oxford

Old Road Campus Research Building, Oxford, OX3 7DQ, United Kingdom

[2] Peter Medawar Building for Pathogen Research, University of Oxford, Oxford, OX1 3SY, United Kingdom

[3] Translational Gastroenterology Unit, Nuffield Department of Medicine, University of Oxford, Oxford, OX3 9DU, United Kingdom

[4] Department of Medical Oncology, Cancer Center, West China Hospital, Sichuan University, Chengdu, 610041, China

[5] Celleron Therapeutics Ltd, Magdalen Centre, Oxford Science Park, Oxford, OX4 4GA, United Kingdom

[~] Each author made an equal contribution

* Corresponding Author email: nick.lathangue@oncology.ox.ac.uk

Running title: HDAC inhibitors augment the anti-covid19 immune response

Keywords: HDAC inhibitors, MHC genes, covid19 spike protein, T and B cells

- Supplementary figures and legends S1-S18
- Supplementary dataset 1

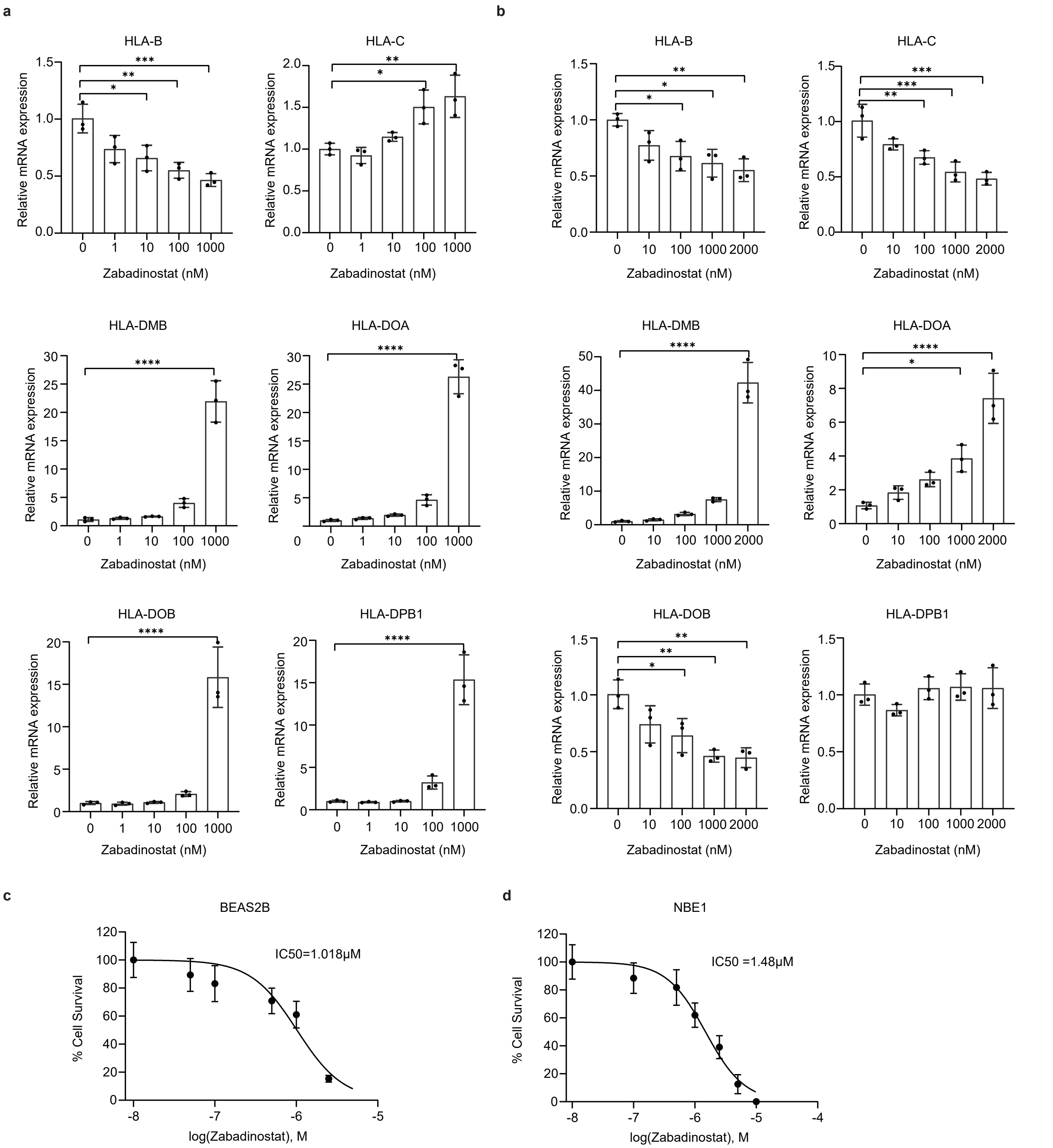

a
HLA-B
HLA-C
HLA-DMB
HLA-DOA
HLA-DOB
HLA-DPB1
Relative mRNA expression
Zabadinostat (nM)
b
HLA-B
HLA-C
HLA-DMB
HLA-DOA
HLA-DOB
HLA-DPB1
Relative mRNA expression
Zabadinostat (nM)
c
BEAS2B
IC50=1.018μM
% Cell Survival
log(Zabadinostat), M
d
NBE1
IC50 =1.48μM
% Cell Survival
log(Zabadinostat), M

Supplementary Figure 1. Expression of MHC class I and class II genes upon zabadinostat treatment. a) Quantitative reverse transcription PCR (qRT-PCR) of MHC class I and class II genes in BEAS2B cells treated for 3 days with 1, 10, 100, 1000 nM zabadinostat or DMSO control; n=3; results presented as mean values +/-SD; one-way ANOVA; **b)** Quantitative reverse transcription PCR (qRT-PCR) of MHC class I and class II genes in NBE1 cells treated for 3 days with 10, 100, 1000, 2000 nM zabadinostat or DMSO control; n=3; results presented as mean values +/-SD; one-way ANOVA; **c)** An IC50 graph from MTT assays performed after treating BEAS2B cells for 3 days with increasing concentrations of zabadinostat; n=3, results presented as mean values +/-SD; **d)** An IC50 graph from MTT assays performed after treating NBE1 cells for 3 days with increasing concentrations of zabadinostat; n=3, results presented as mean values +/-SD.

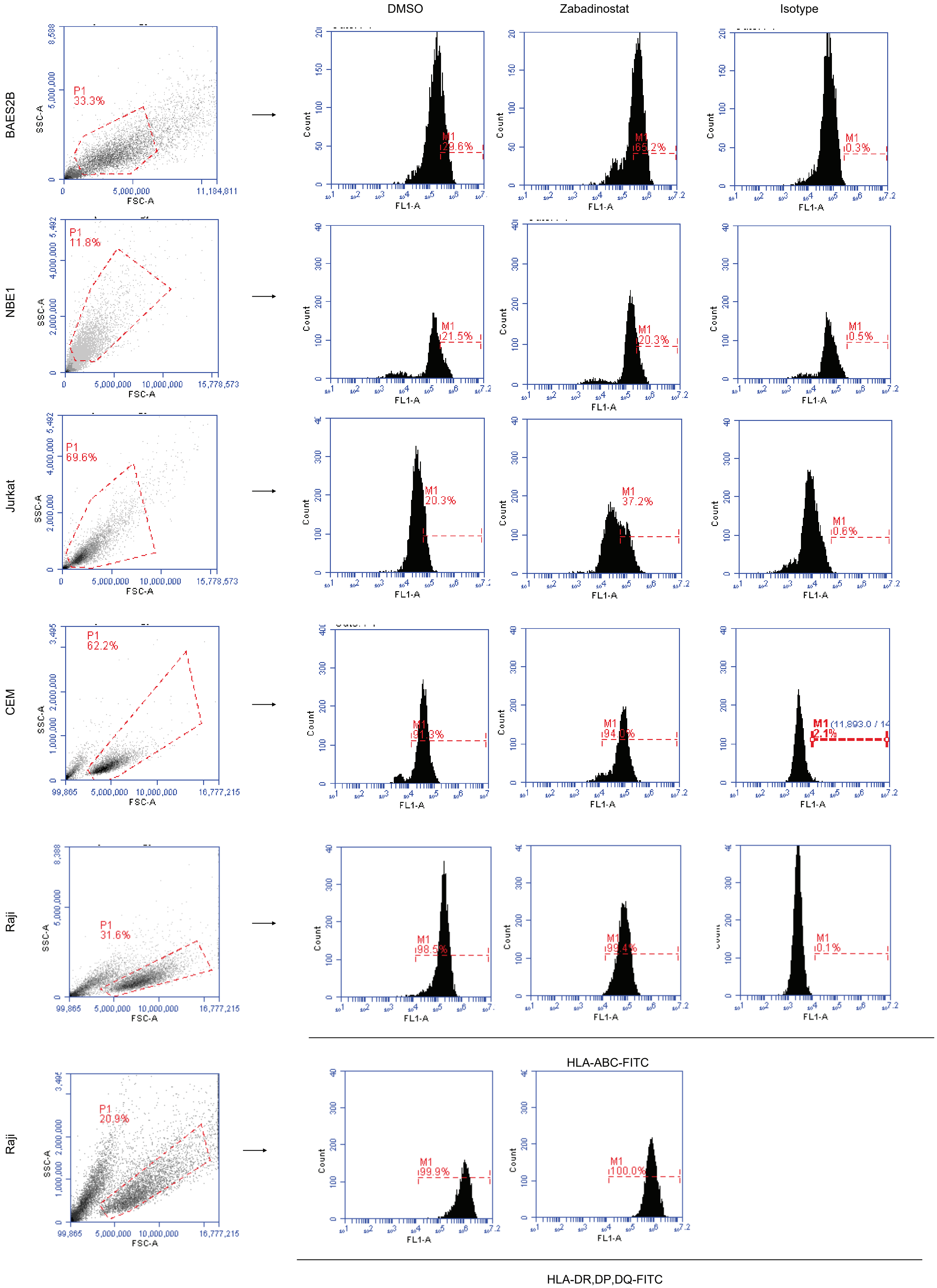

DMSO
Zabadinostat
Isotype
BAES2B
P1
33.3%
M1
29.6%
M1
65.2%
M1
0.3%
NBE1
P1
11.8%
M1
21.5%
M1
20.3%
M1
0.5%
Jurkat
P1
69.6%
M1
20.3%
M1
37.2%
M1
0.6%
CEM
P1
62.2%
M1
91.3%
M1
94.0%
M1
2.1%
Raji
P1
31.6%
M1
98.5%
M1
99.4%
M1
0.1%
HLA-ABC-FITC
Raji
P1
20.9%
M1
99.9%
M1
100.0%
HLA-DR,DP,DQ-FITC
SSC-A
FSC-A
Count
FL1-A

Supplementary Figure 2. Representative flow cytometry plots and gating strategy for the results presented in the Figure 1c-d and Supplementary Figure 3a-c.

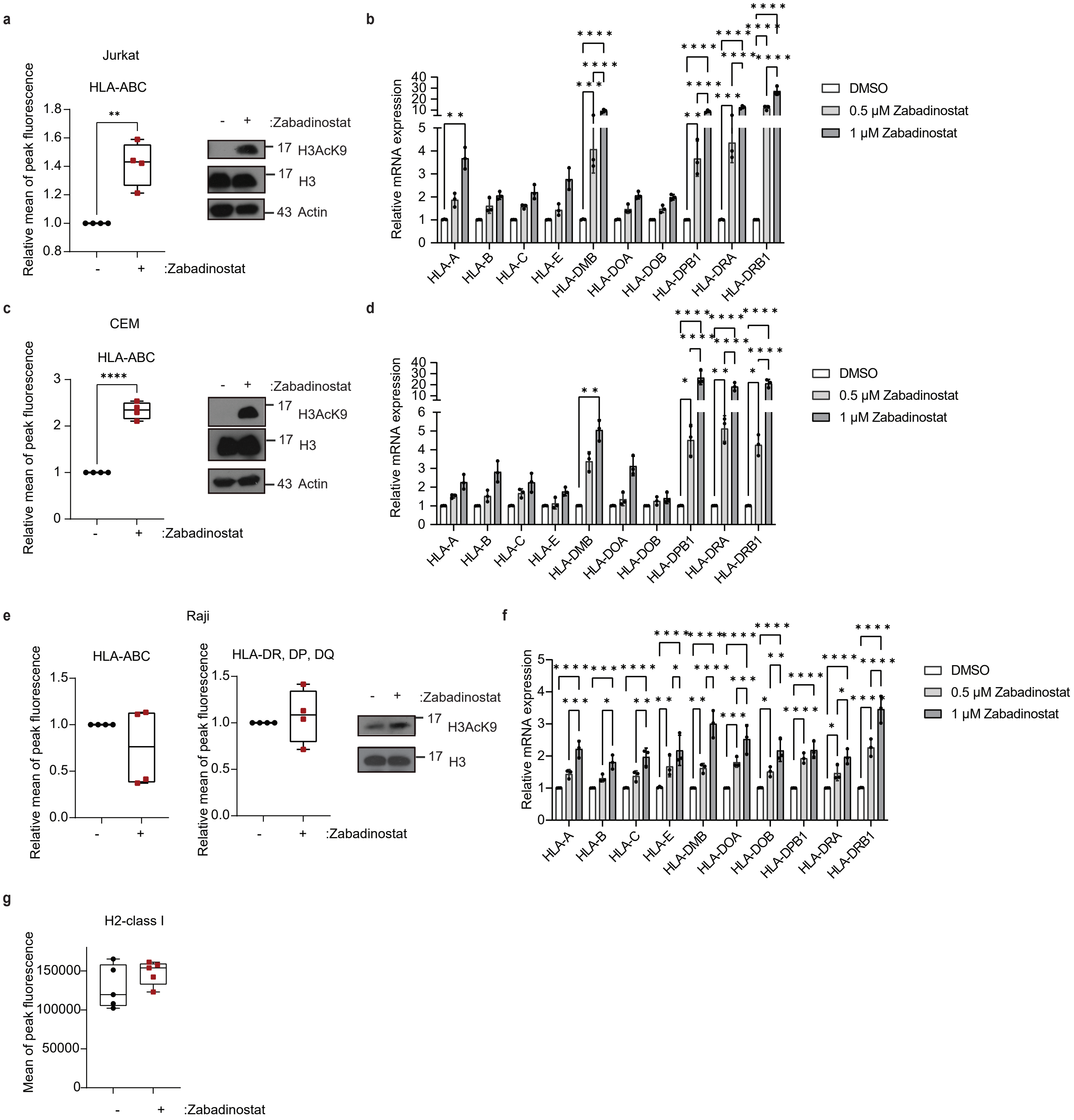
a
Jurkat
HLA-ABC
Relative mean of peak fluorescence
1.8
1.6
1.4
1.2
1.0
0.8
**
-
+
:Zabadinostat
H3AcK9
H3
Actin
17
17
43
b
Relative mRNA expression
DMSO
0.5 μM Zabadinostat
1 μM Zabadinostat
HLA-A
HLA-B
HLA-C
HLA-E
HLA-DMB
HLA-DOA
HLA-DOB
HLA-DPB1
HLA-DRA
HLA-DRB1
c
CEM
HLA-ABC
****
d
e
Raji
HLA-ABC
HLA-DR, DP, DQ
f
g
H2-class I
Mean of peak fluorescence
150000
100000
50000
0

Supplementary Figure 3. Expression of MHC class I and class II genes upon zabadinostat treatment. a) flow cytometry analysis of extracellular MHC class I proteins in Jurkat cells treated for 2 days with 1 µM zabadinostat or DMSO control, n=4, results presented as mean values +/-SD, Student's t test; The acetylation mark (H3AcK9) was detected by immunoblotting; **b)** Quantitative reverse transcription PCR (qRT-PCR) of MHC class I and class II genes in Jurkat cells treated for 2 days with 0.5 and 1 µM zabadinostat or DMSO control; n=3; results presented as mean values +/-SD; one-way ANOVA; **c)** flow cytometry analysis of extracellular MHC class I proteins in CEM cells treated for 2 days with 1 µM zabadinostat or DMSO control, n=4, results presented as mean values +/-SD, Student's t test; The acetylation mark (H3AcK9) was detected by immunoblotting; **d)** Quantitative reverse transcription PCR (qRT-PCR) of MHC class I and class II genes in CEM cells treated for 2 days with 0.5 and 1 µM zabadinostat or DMSO control; n=3; results presented as mean values +/-SD; one-way ANOVA; **e)** flow cytometry analysis of extracellular MHC class I and II proteins in Raji cells treated for 2 days with 1 µM zabadinostat or DMSO control, n=4; The acetylation mark (H3AcK9) was detected by immunoblotting; **f)** Quantitative reverse transcription PCR (qRT-PCR) of MHC class I and class II genes in Raji cells treated for 2 days with 0.5 and 1 µM zabadinostat or DMSO control; n=3; results presented as mean values +/-SD; one-way ANOVA; **g)** flow cytometry analysis of extracellular MHC class I in bone marrow (collected from C57BL/6)-derived dendritic cells (CD11c+/CD86+) (from Figure 3a) treated with 1 µM zabadinostat or DMSO control for 48 hours; n=5.

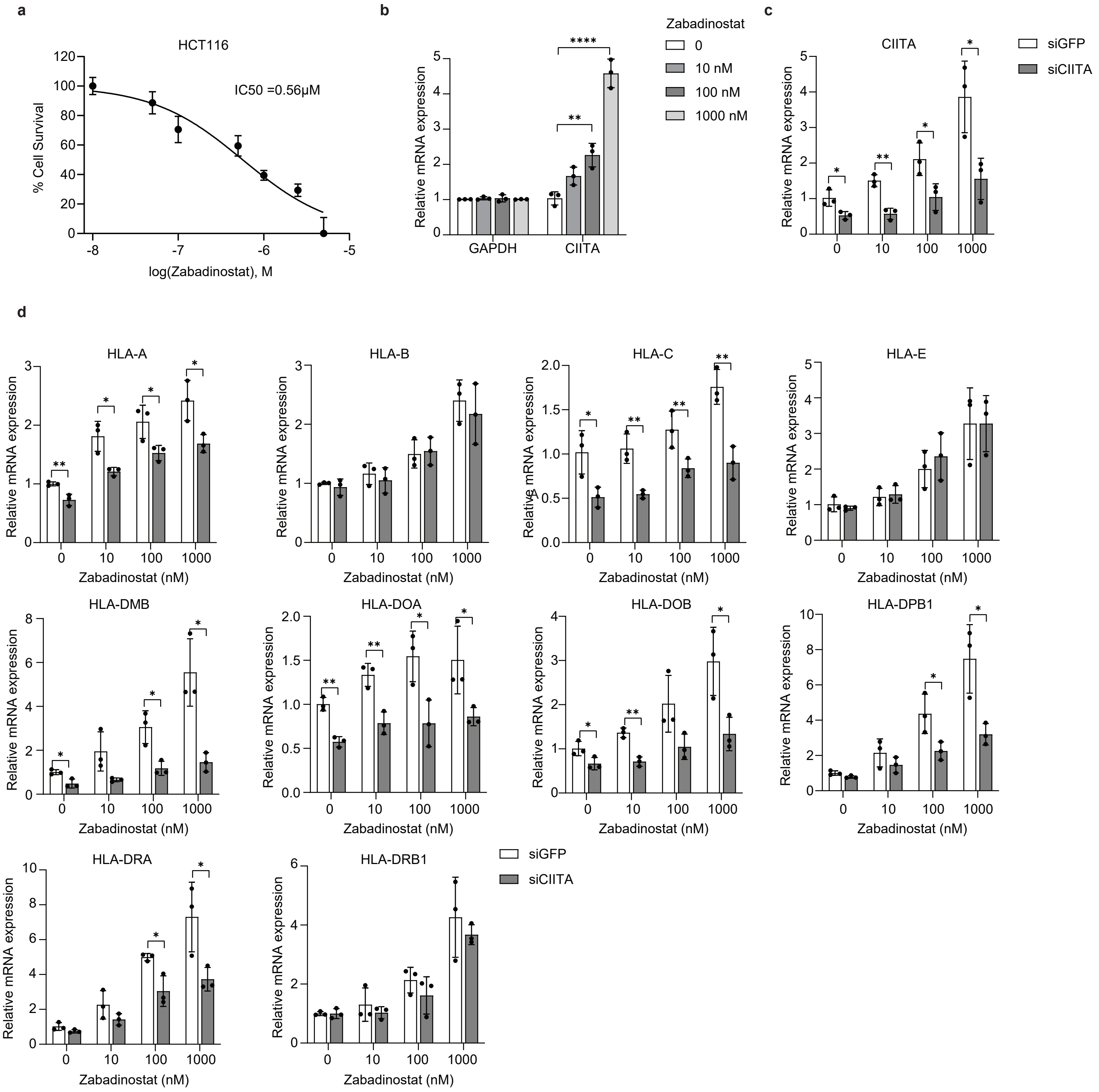
a
HCT116
IC50 =0.56μM
% Cell Survival
log(Zabadinostat), M
b
Zabadinostat
0
10 nM
100 nM
1000 nM
Relative mRNA expression
GAPDH
CIITA
c
CIITA
siGFP
siCIITA
d
HLA-A
HLA-B
HLA-C
HLA-E
HLA-DMB
HLA-DOA
HLA-DOB
HLA-DPB1
HLA-DRA
HLA-DRB1
Zabadinostat (nM)
siGFP
siCIITA

Supplementary Figure 4. CIITA is a positive regulator of MHC class I and class II gene expression in HCT116 cells. a) An IC50 graph from MTT assays performed after treating HCT116 cells for 3 days with increasing concentrations of zabadinostat; n=3, results presented as mean values +/-SD; **b)** Quantitative reverse transcription PCR (qRT-PCR) of CIITA in HCT116 cells treated for 3 days with 10, 100, 1000 nM zabadinostat or DMSO control; n=3; results presented as mean values +/-SD; one-way ANOVA; **c)** The expression of CIITA after siRNA treatment were detected by qRT-PCR; results presented as mean values +/-SD; one-way ANOVA; **d)** Quantitative reverse transcription PCR (qRT-PCR) of MHC class I and class II genes in HCT116 cells treated for 2 days with 50nM siCIITA and for 3 days with 10, 100, 1000 nM zabadinostat or DMSO control; n=3; results presented as mean values +/-SD; one-way ANOVA;.

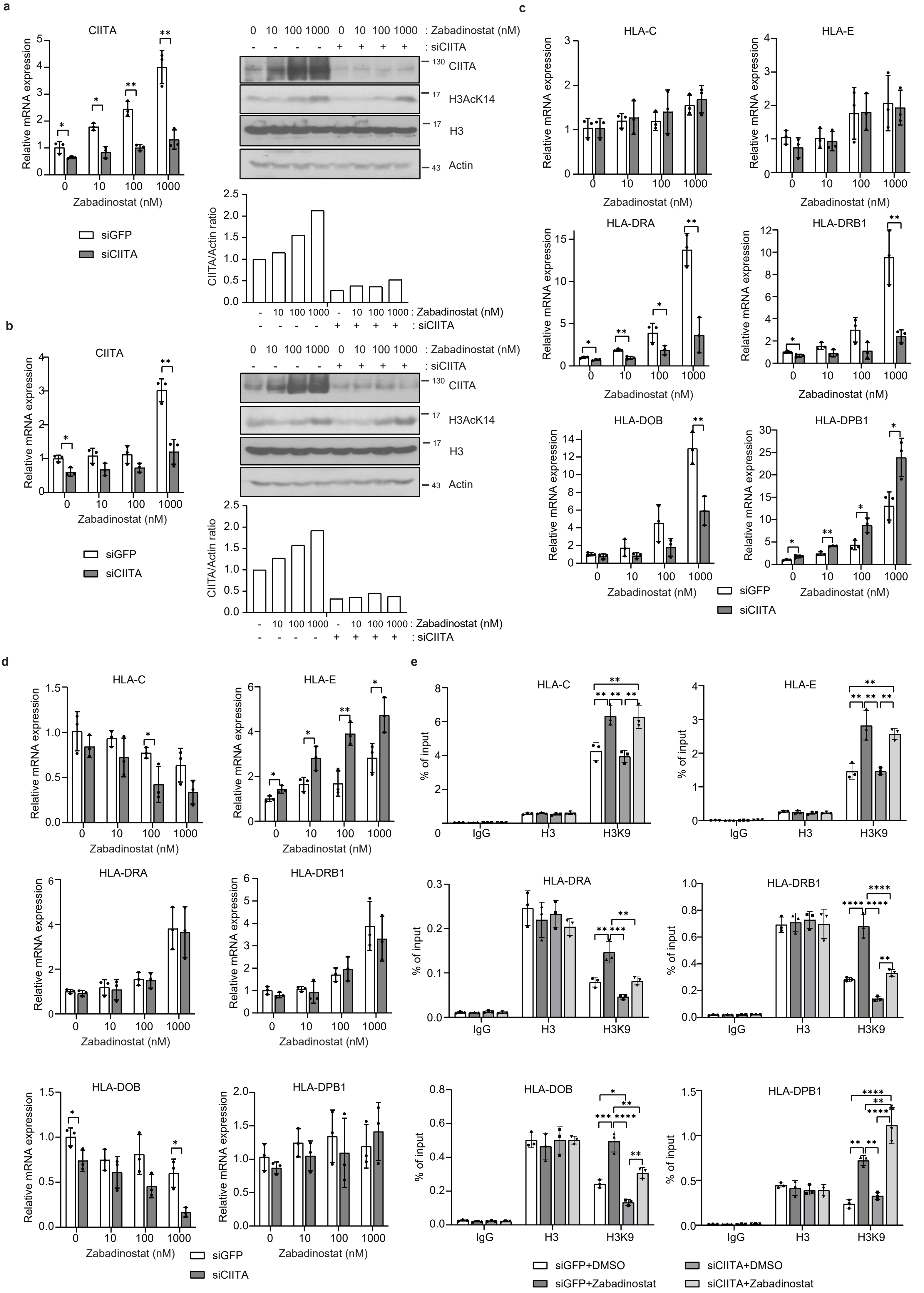
a
b
c
d
e
CIITA
Relative mRNA expression
Zabadinostat (nM)
siGFP
siCIITA
0 10 100 1000 : Zabadinostat (nM)
- - - - + + + + : siCIITA
130 CIITA
17 H3AcK14
17 H3
43 Actin
CIITA/Actin ratio
- 10 100 1000 - 10 100 1000 : Zabadinostat (nM)
HLA-C
HLA-E
HLA-DRA
HLA-DRB1
HLA-DOB
HLA-DPB1
% of input
IgG
H3
H3K9
siGFP+DMSO
siGFP+Zabadinostat
siCIITA+DMSO
siCIITA+Zabadinostat

Supplementary Figure 5. CIITA is a positive regulator of MHC class I and class II genes in BEAS2B and NBE1 cells. a) The expression of CIITA after siRNA treatment were detected by qRT-PCR, n=3; results presented as mean values +/-SD; one-way ANOVA; The CIITA protein level and acetylation mark (H3AcK14) were detected by immunoblotting and quantified; **b)** The expression of CIITA after siRNA treatment was detected by qRT-PCR, n=3; results presented as mean values +/-SD; one-way ANOVA; The CIITA protein level and acetylation mark (H3AcK14) were detected by immunoblotting and quantified; **c)** Quantitative reverse transcription PCR (qRT-PCR) of MHC class I and class II genes in BEAS2B cells treated for 2 days with 50nM siCIITA and for 3 days with 10, 100, 1000 nM zabadinostat or DMSO control; n=3; results presented as mean values +/-SD; one-way ANOVA; **d)** Quantitative reverse transcription PCR (qRT-PCR) of MHC class I and class II genes in NBE1 cells treated for 2 days with 50nM siCIITA and for 3 days with 10, 100, 1000 nM zabadinostat or DMSO control; n=3; results presented as mean values +/-SD; one-way ANOVA; **e)** Histone H3 and H3AcK9 ChIP on MHC gene promoters in BEAS2B cells treated for 2 days with 50nM siCIITA and for 3 days with 1000 nM zabadinostat or DMSO control; n=3; results presented as mean values +/-SD; one-way ANOVA.

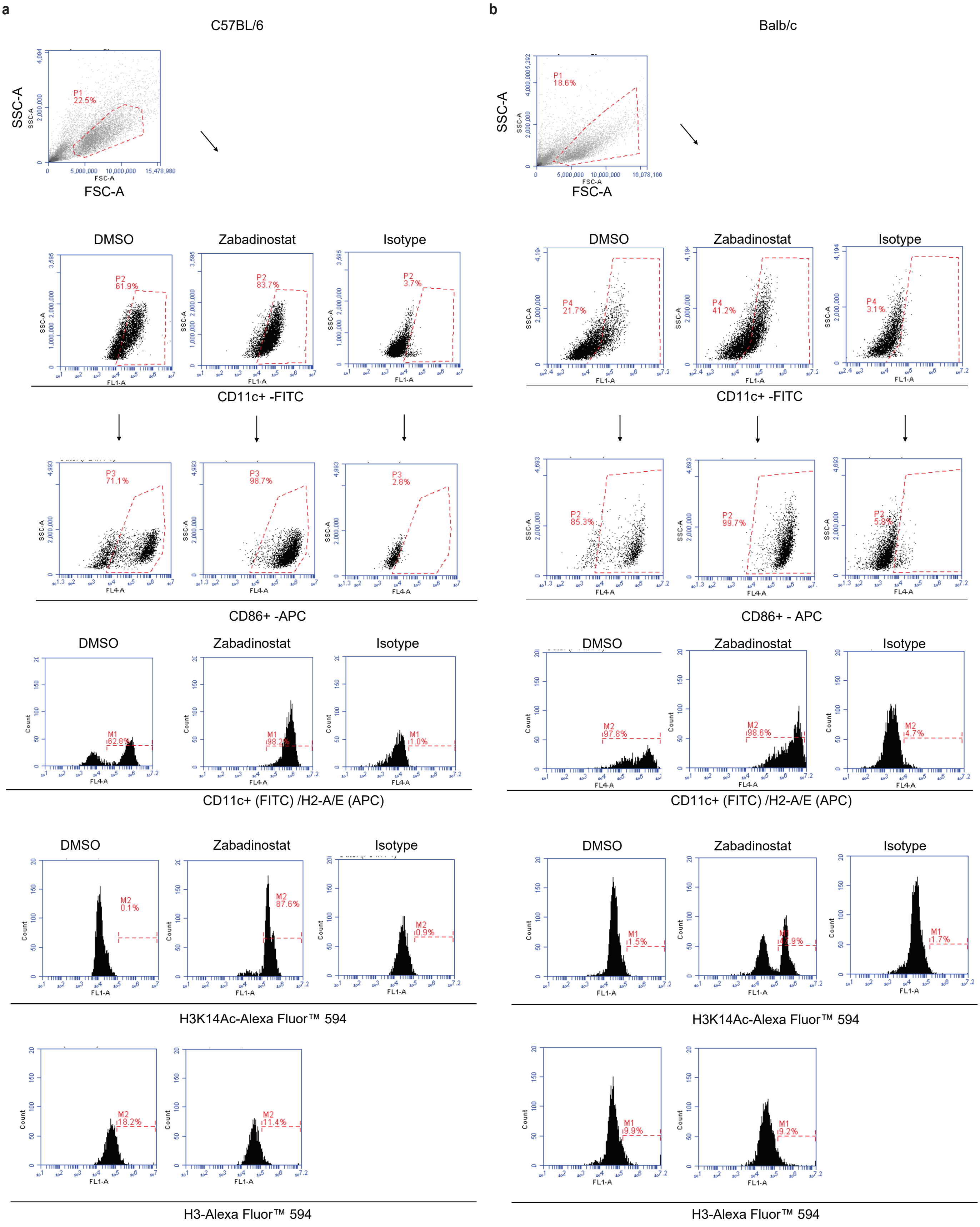
a
C57BL/6
SSC-A
FSC-A
P1
22.5%
DMSO
Zabadinostat
Isotype
P2
61.9%
83.7%
3.7%
CD11c+ -FITC
P3
71.1%
98.7%
2.8%
CD86+ -APC
M1
62.8%
98.2%
1.0%
CD11c+ (FITC) /H2-A/E (APC)
M2
0.1%
87.6%
0.9%
H3K14Ac-Alexa Fluor™ 594
18.2%
11.4%
H3-Alexa Fluor™ 594
b
Balb/c
P1
18.6%
P4
21.7%
41.2%
3.1%
P2
85.3%
99.7%
97.8%
98.6%
4.7%
CD86+ - APC
1.5%
1.7%
9.9%
9.2%
Count
FL1-A
FL4-A

Supplementary Figure 6. Representative flow cytometry plots and gating strategy (a-b) for the results presented in the Figure 3a and 3c.

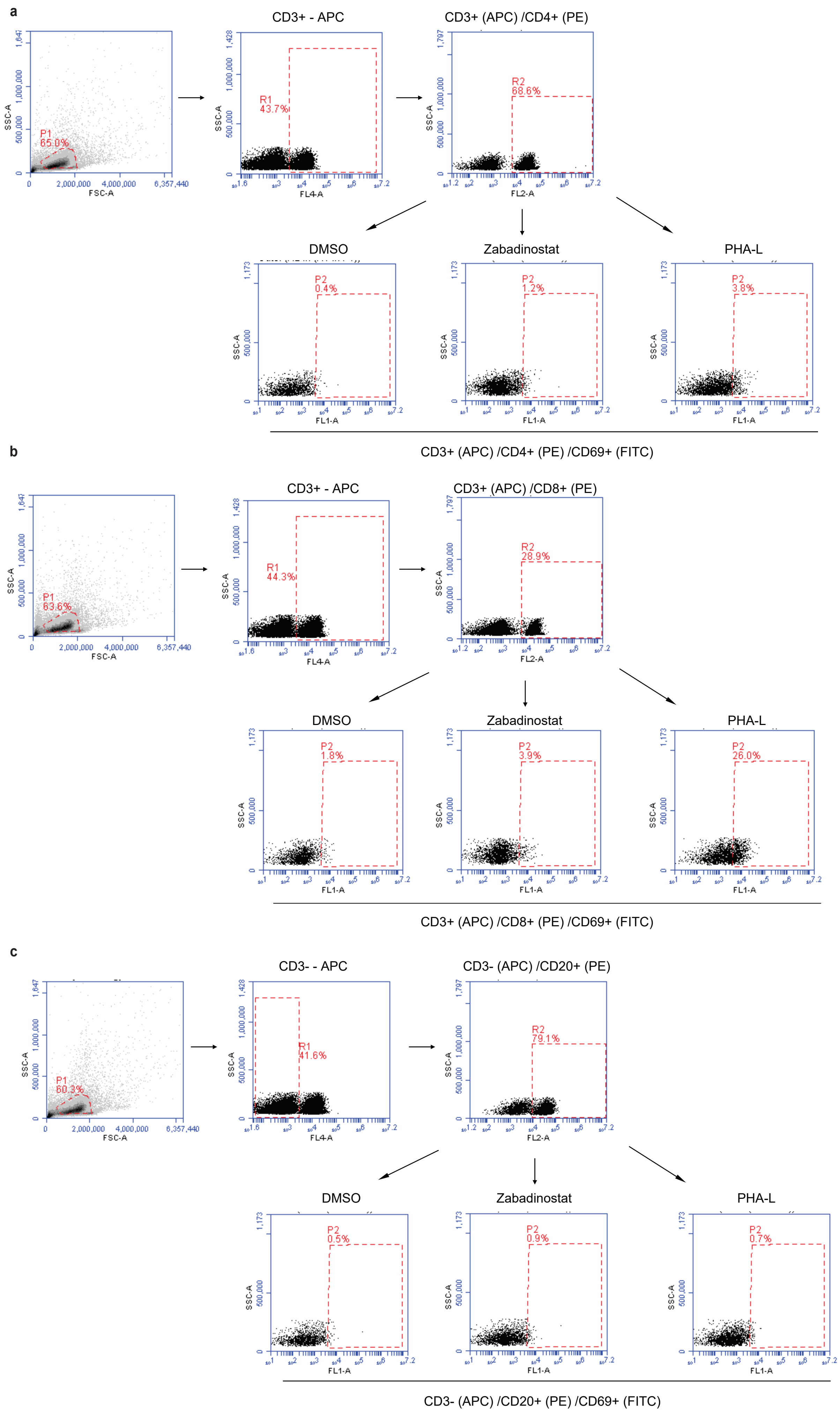

a
CD3+ - APC
CD3+ (APC) /CD4+ (PE)
P1
65.0%
SSC-A
FSC-A
R1
43.7%
FL4-A
R2
68.6%
FL2-A
DMSO
Zabadinostat
PHA-L
P2
0.4%
P2
1.2%
P2
3.8%
FL1-A
CD3+ (APC) /CD4+ (PE) /CD69+ (FITC)
b
CD3+ - APC
CD3+ (APC) /CD8+ (PE)
P1
63.6%
R1
44.3%
R2
28.9%
DMSO
Zabadinostat
PHA-L
P2
1.8%
P2
3.9%
P2
26.0%
CD3+ (APC) /CD8+ (PE) /CD69+ (FITC)
c
CD3- - APC
CD3- (APC) /CD20+ (PE)
P1
60.3%
R1
41.6%
R2
79.1%
DMSO
Zabadinostat
PHA-L
P2
0.5%
P2
0.9%
P2
0.7%
CD3- (APC) /CD20+ (PE) /CD69+ (FITC)

Supplementary Figure 7. Representative flow cytometry plots and gating strategy (a-c) for the results presented in the Figure 3d.

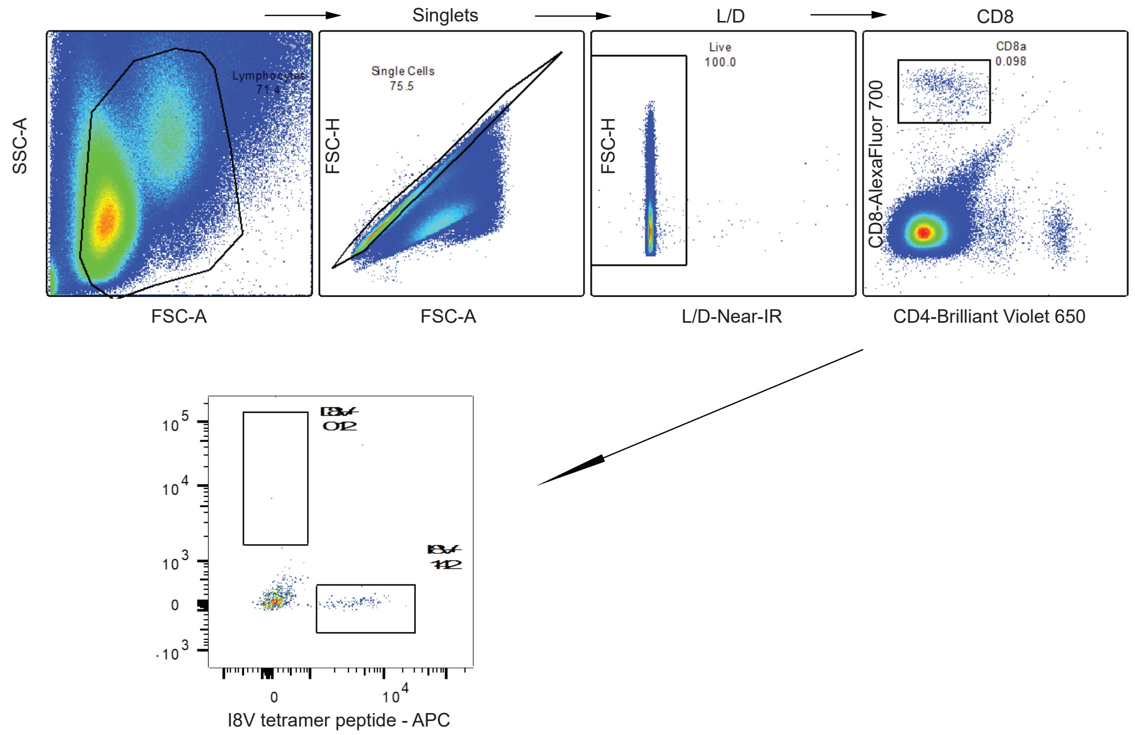

Singlets
L/D
CD8
SSC-A
FSC-A
Single Cells
75.5
FSC-H
FSC-A
Live
100.0
FSC-H
L/D-Near-IR
CD8a
0.098
CD8-AlexaFluor 700
CD4-Brilliant Violet 650
10^5
10^4
10^3
0
-10^3
0
10^4
I8V tetramer peptide - APC

Supplementary Figure 8. Representative flow cytometry plots and gating strategy for the results presented in the Figure 4c-d.

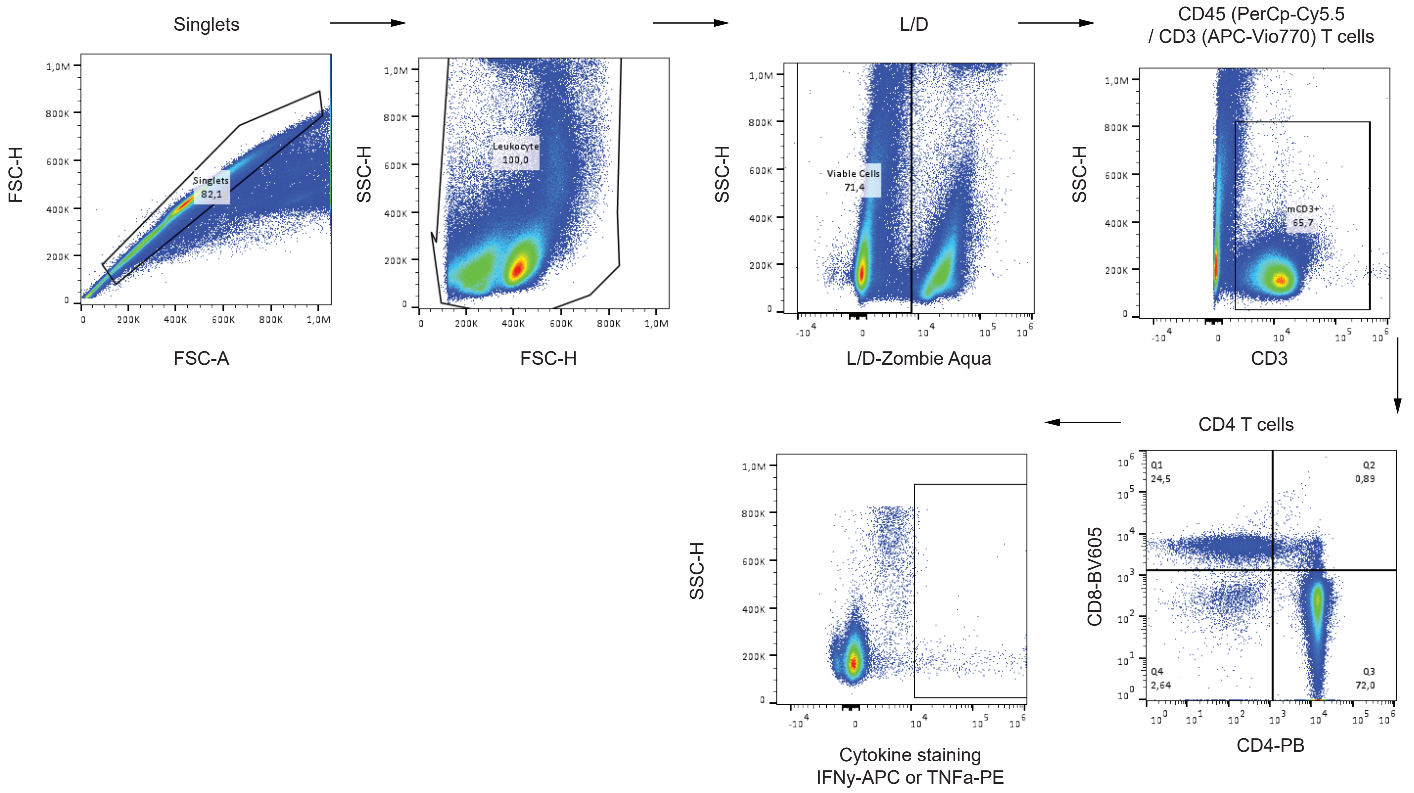
Singlets
Singlets 82,1
FSC-H
FSC-A
Leukocyte 100,0
SSC-H
FSC-H
L/D
Viable Cells 71,4
SSC-H
L/D-Zombie Aqua
CD45 (PerCp-Cy5.5 / CD3 (APC-Vio770) T cells
mCD3+ 65,7
SSC-H
CD3
CD4 T cells
Q1 24,5
Q2 0,89
Q3 72,0
Q4 2,64
CD8-BV605
CD4-PB
SSC-H
Cytokine staining
IFNy-APC or TNFa-PE

Supplementary Figure 9. Representative flow cytometry plots and gating strategy for the results presented in the Figure 5c and Supplementary Figure 15.

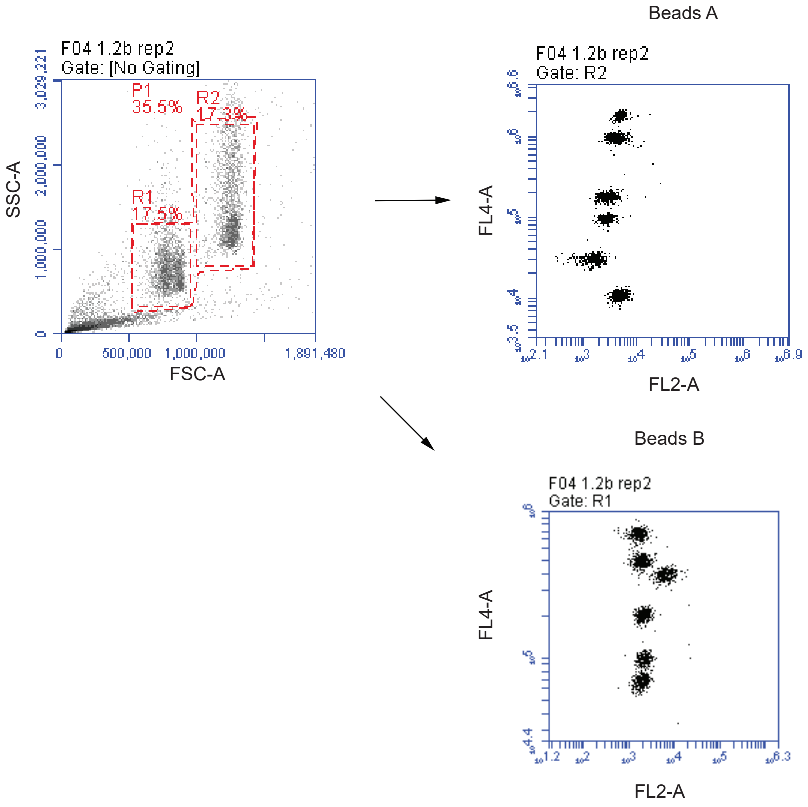

F04 1.2b rep2
Gate: [No Gating]
P1
35.5%
R2
17.3%
R1
17.5%
SSC-A
FSC-A
0
500,000
1,000,000
1,891,480
1,000,000
2,000,000
3,029,221
Beads A
F04 1.2b rep2
Gate: R2
FL4-A
FL2-A
Beads B
F04 1.2b rep2
Gate: R1
FL4-A
FL2-A

Supplementary Figure 10. Representative flow cytometry plots and gating strategy for the results presented in the Figure 5d and Supplementary Figure 11.

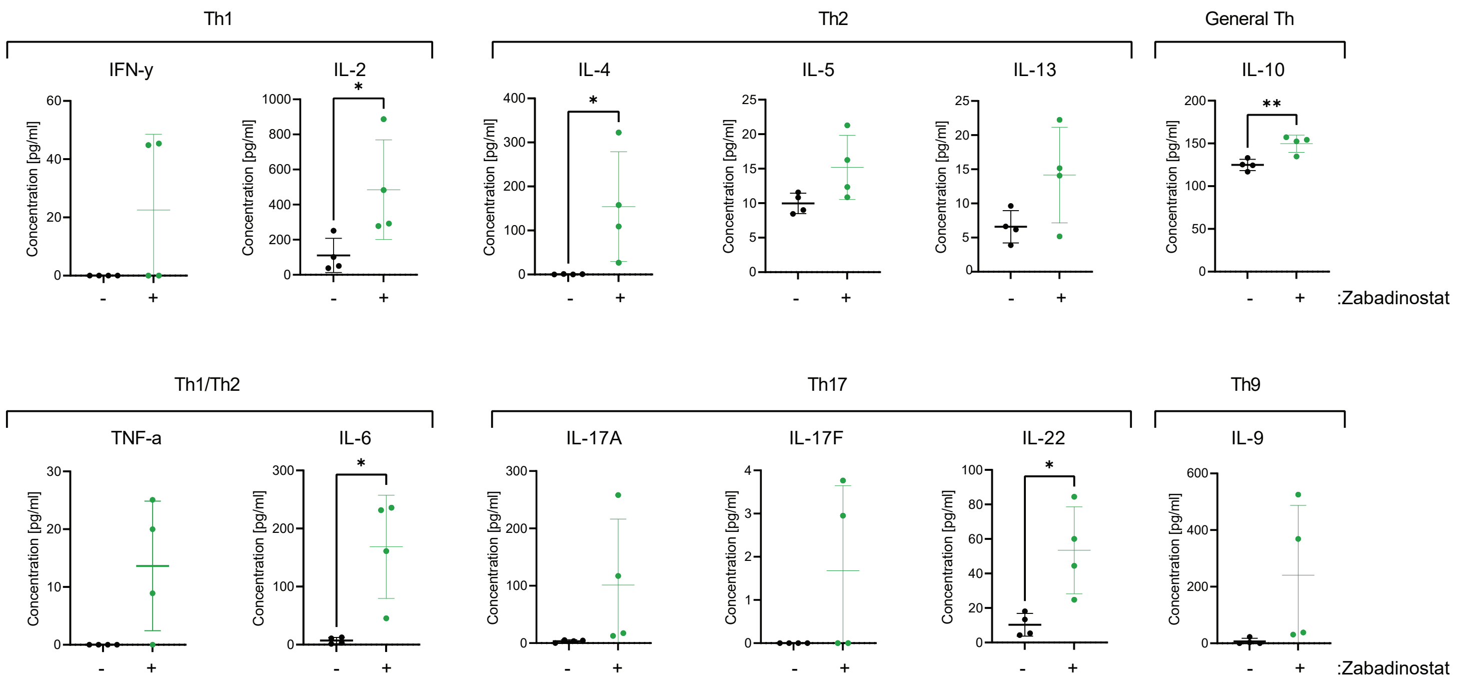
Th1
IFN-y
IL-2
Th2
IL-4
IL-5
IL-13
General Th
IL-10
Th1/Th2
TNF-a
IL-6
Th17
IL-17A
IL-17F
IL-22
Th9
IL-9
Concentration [pg/ml]
:Zabadinostat

Supplementary Figure 11. Zabadinostat increases cytokine release. Analysis of panel of Th1, Th2, Th1/Th2, General Th, Th17, and Th9 cytokines in serum collected from mice treated with zabadinostat and the control group; Balb/c mice were treated with orally administrated zabadinostat at 25 mg/kg for 14 days (5 days on/2 days off) or vehicle only; n=4 per group; results presented as mean values +/-SD; Student's t test.

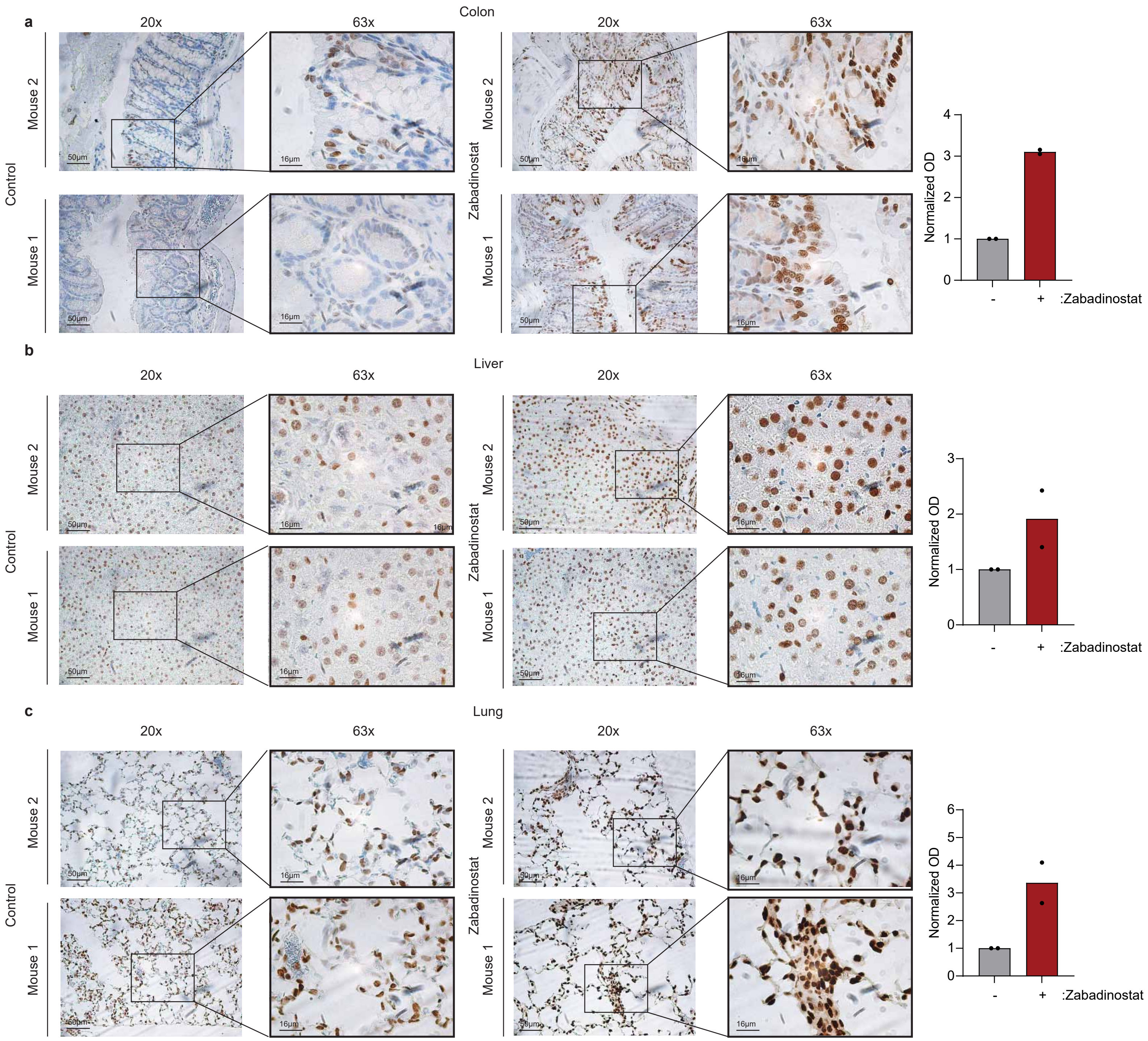
a
Colon
20x
63x
Control
Mouse 2
Mouse 1
Zabadinostat
50μm
16μm
Normalized OD
0
1
2
3
4
-
+
:Zabadinostat
b
Liver
20x
63x
Control
Mouse 2
Mouse 1
Zabadinostat
50μm
16μm
Normalized OD
0
1
2
3
-
+
:Zabadinostat
c
Lung
20x
63x
Control
Mouse 2
Mouse 1
Zabadinostat
50μm
16μm
Normalized OD
0
1
2
3
4
5
6
-
+
:Zabadinostat

Supplementary Figure 12. Zabadinostat efficacy in different tissues. Representative examples of immunohistochemical staining of H3AcK9 in colon **(a)**, liver **(b)**, and lung **(c)** collected from Balb/c mice at 14 days treated with 25 mg/kg zabadinostat and non-treated control (5 days on/2 days off). Original magnification: 20x, scale bar, 50 µm; and 63x; scale bar, 16 µm. n=2; results were quantified by ImageJ Fiji software and normalised optical density was presented as a mean +/- SD. Statistical analysis was performed using two-tailed, unpaired Student's t-test with GraphPad Prism 8 software, n=2.

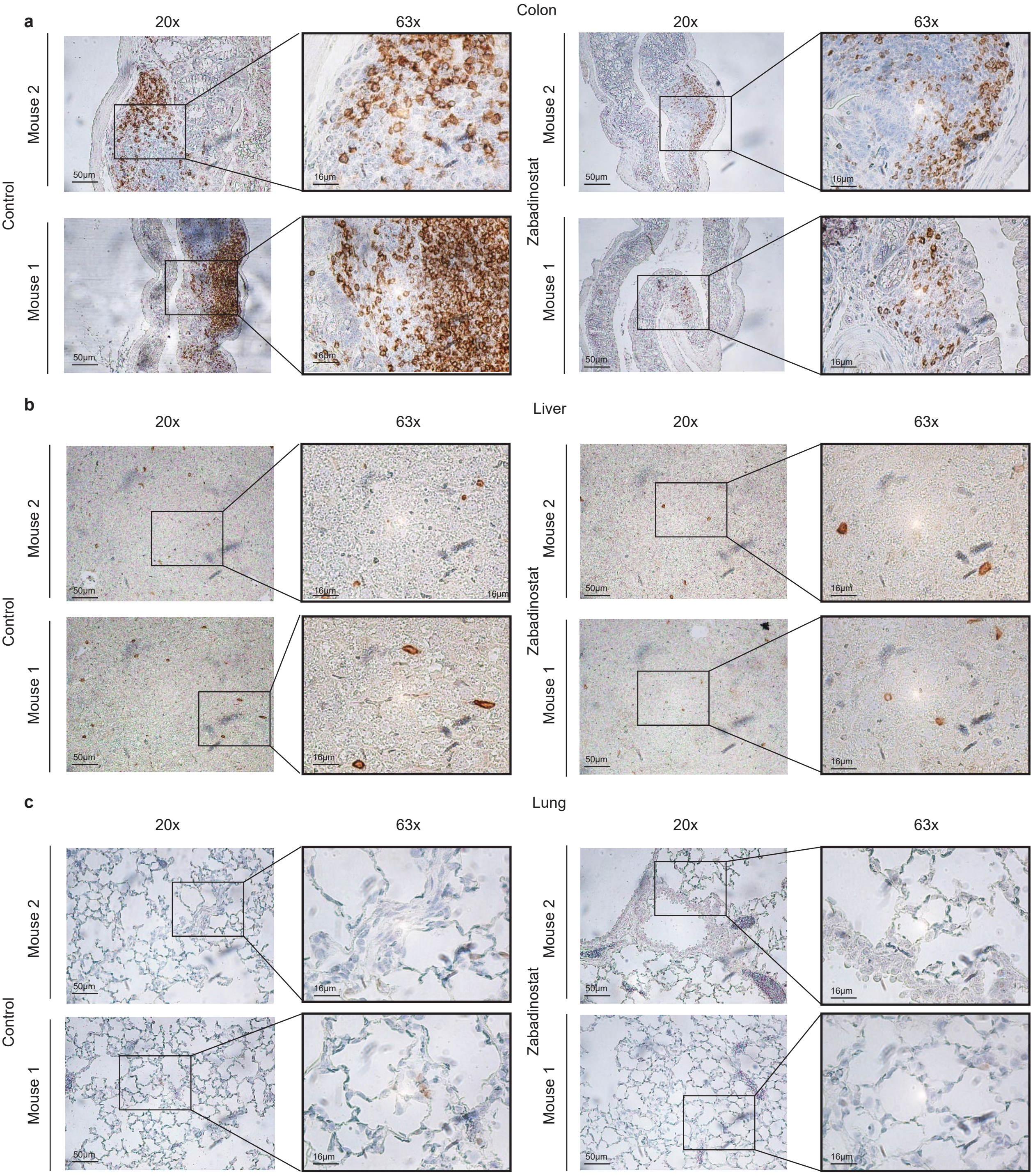

a
Colon
20x
63x
Control
Mouse 2
Mouse 1
Zabadinostat
50μm
16μm
b
Liver
c
Lung

Supplementary Figure 13. CD4 T cell infiltration in different tissues upon zabadinostat treatment. Representative examples of immunohistochemical staining of CD4 T cells in colon **(a)**, liver **(b)**, and lung **(c)** collected from Balb/c mice at 14 days treated with 25 mg/kg zabadinostat and non-treated control (5 days on/2 days off). Original magnification: 20x, scale bar, 50 μm; and 63x; scale bar, 16 μm. n=2.

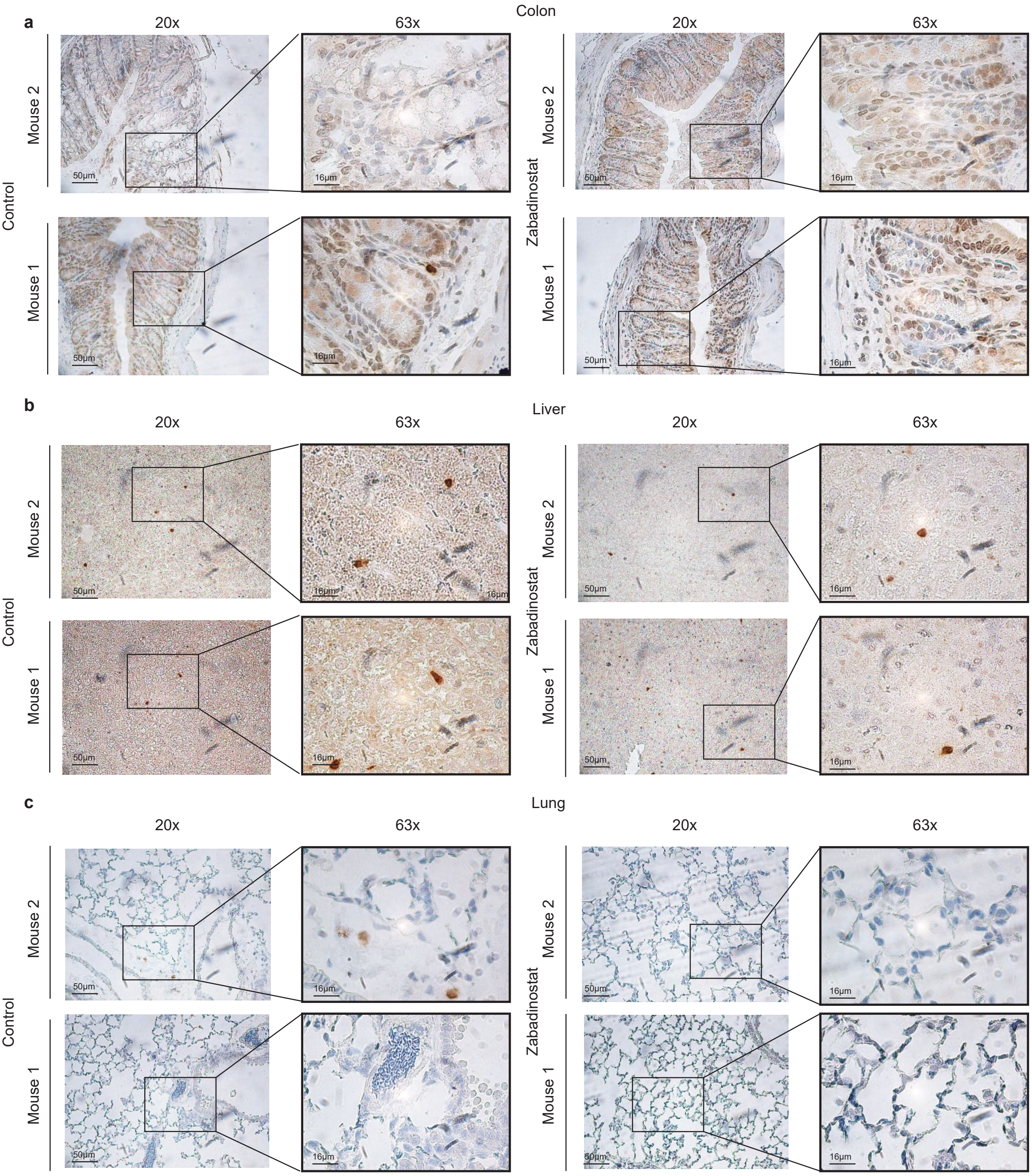
a
Colon
b
Liver
c
Lung
20x
63x
Control
Zabadinostat
Mouse 2
Mouse 1
50µm
16µm

Supplementary Figure 14. CD8 T cell infiltration in different tissues upon zabadinostat treatment. Representative examples of immunohistochemical staining of CD8 T cells in colon **(a)**, liver **(b)**, and lung **(c)** collected from Balb/c mice at 14 days treated with 25 mg/kg zabadinostat and non-treated control (5 days on/2 days off). Original magnification: 20x, scale bar, 50 μm; and 63x; scale bar, 16 μm. n=2.

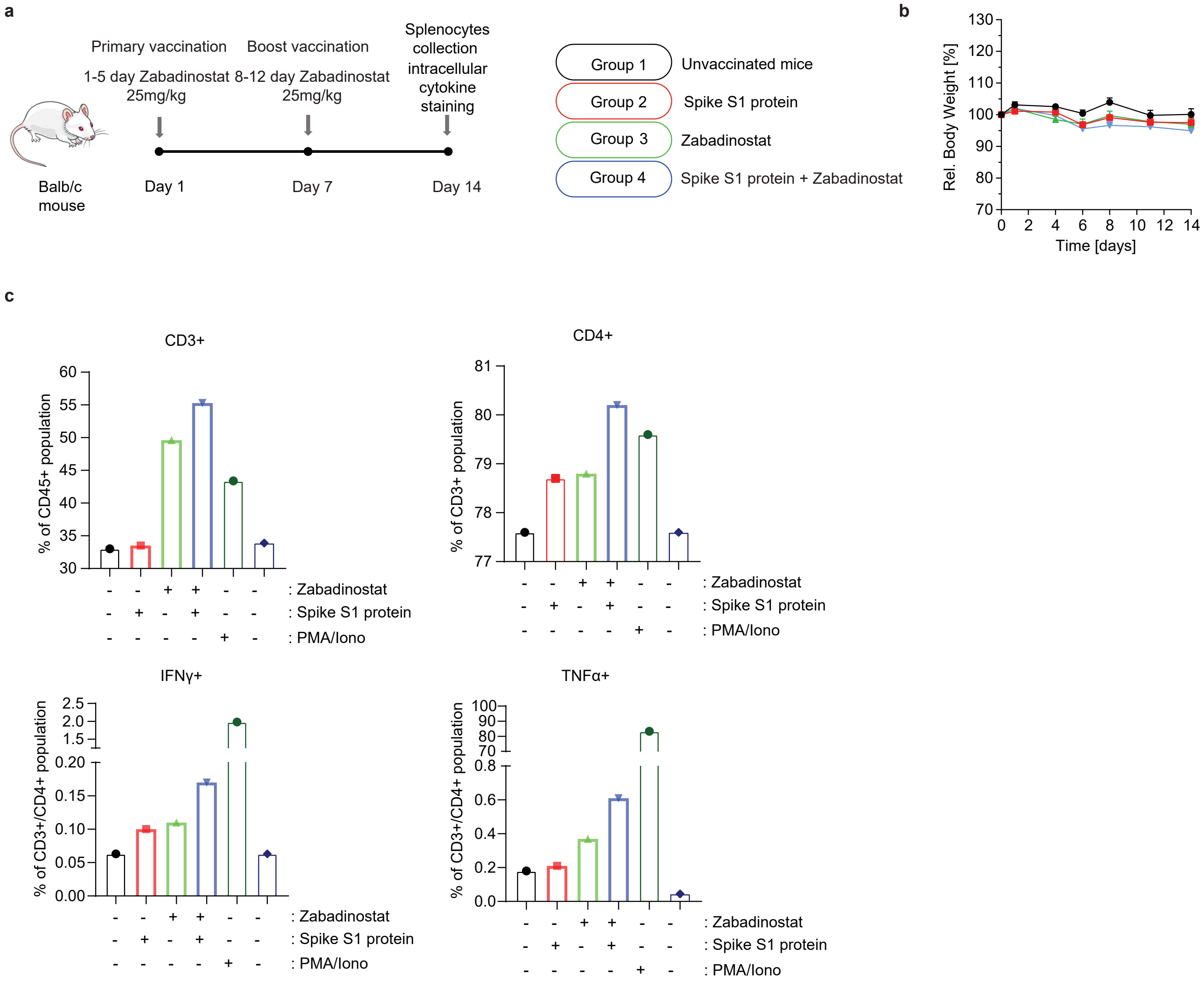
a
Primary vaccination
Boost vaccination
Splenocytes collection intracellular cytokine staining
1-5 day Zabadinostat 25mg/kg
8-12 day Zabadinostat 25mg/kg
Balb/c mouse
Day 1
Day 7
Day 14
Group 1
Unvaccinated mice
Group 2
Spike S1 protein
Group 3
Zabadinostat
Group 4
Spike S1 protein + Zabadinostat
b
Rel. Body Weight [%]
Time [days]
c
CD3+
% of CD45+ population
CD4+
% of CD3+ population
IFNγ+
% of CD3+/CD4+ population
TNFα+
% of CD3+/CD4+ population
: Zabadinostat
: Spike S1 protein
: PMA/Iono

Supplementary Figure 15. **Zabadinostat enhances the T cell response to covid spike S1 protein. a)** Schematic representation of the experiment. Balb/c mice were treated with orally administrated zabadinostat at 25 mg/kg for 14 days (5 days on/2 days off) and intravenous spike S1 protein (days 1 and 7) with respect to vehicle only control; n=4 per group; **b)** relative body weight representation of treated and non-treated mice; results presented as mean values +/-SD; **c)** General CD3 positive T cell activation, CD4 positive T cells, and IFNγ, TNFα intracellular cytokine staining (within CD4 positive cell population) measurements by flow cytometry; measurements were performed in pooled splenocytes from 4 mice; also, we noticed similar percentage of CD8 positive T cells in control and zabadinostat treated groups (18.4% and 16%). Noteworthy, viability of the splenocytes was similar in zabadinostat treatment compare to untreated control (67.3% and 74.2% viable cells, respectively).

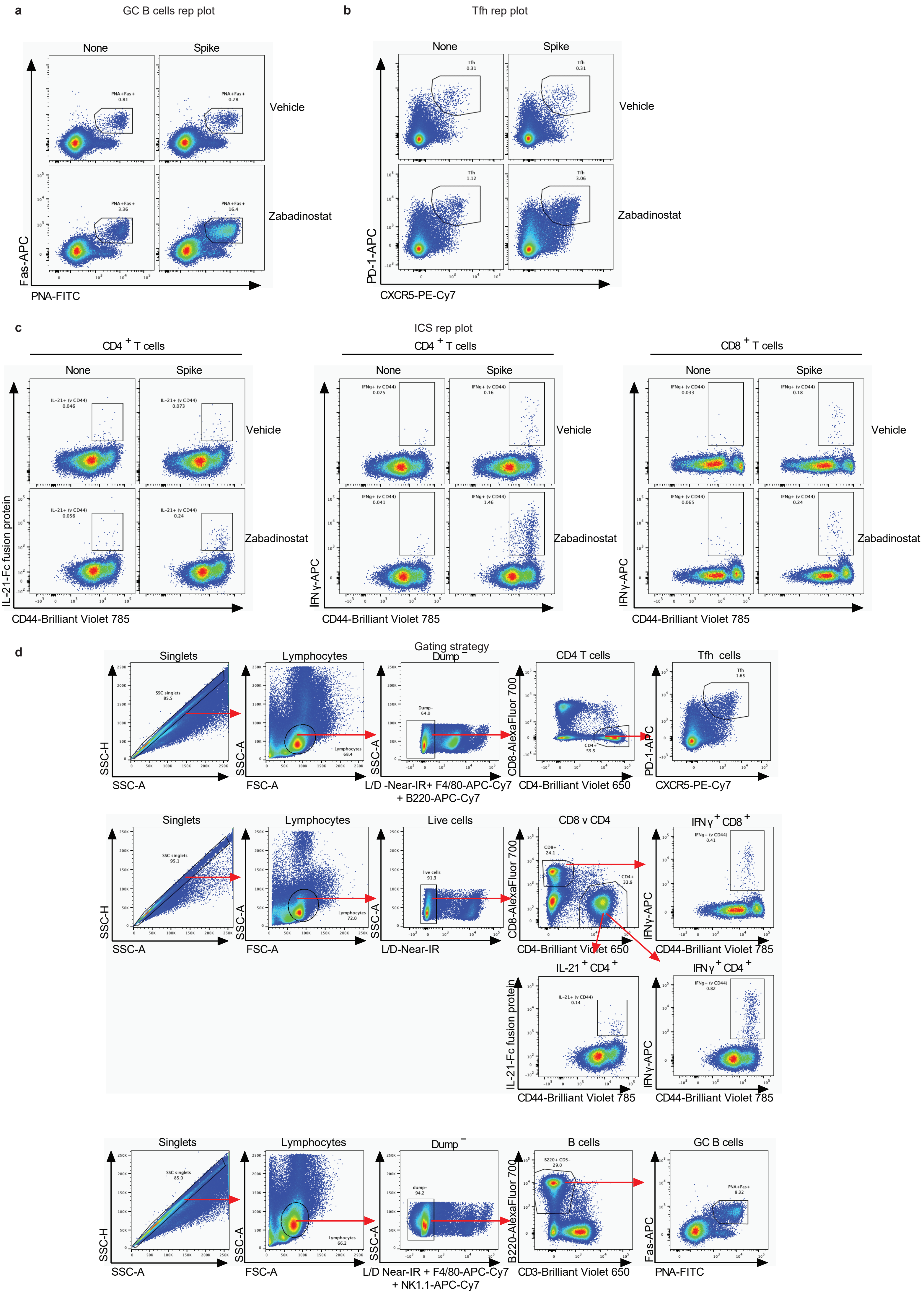
a
GC B cells rep plot
None
Spike
Vehicle
Zabadinostat
PNA+Fas+ 0.81
PNA+Fas+ 0.78
PNA+Fas+ 3.36
PNA+Fas+ 16.4
Fas-APC
PNA-FITC
b
Tfh rep plot
None
Spike
Vehicle
Zabadinostat
Tfh 0.31
Tfh 0.31
Tfh 1.12
Tfh 3.06
PD-1-APC
CXCR5-PE-Cy7
c
ICS rep plot
CD4 + T cells
CD4 + T cells
CD8 + T cells
None
Spike
Vehicle
Zabadinostat
IL-21+ (v CD44) 0.046
IL-21+ (v CD44) 0.073
IL-21+ (v CD44) 0.056
IL-21+ (v CD44) 0.24
IFNg+ (v CD44) 0.025
IFNg+ (v CD44) 0.16
IFNg+ (v CD44) 0.041
IFNg+ (v CD44) 1.46
IFNg+ (v CD44) 0.033
IFNg+ (v CD44) 0.18
IFNg+ (v CD44) 0.065
IFNg+ (v CD44) 0.24
IL-21-Fc fusion protein
IFNγ-APC
CD44-Brilliant Violet 785
d
Gating strategy
Singlets
Lymphocytes
Dump −
CD4 T cells
Tfh cells
SSC singlets 85.5
Lymphocytes 68.4
Dump- 64.0
CD4+ 55.5
Tfh 1.65
SSC-H
SSC-A
FSC-A
L/D -Near-IR+ F4/80-APC-Cy7 + B220-APC-Cy7
CD8-AlexaFluor 700
CD4-Brilliant Violet 650
PD-1-APC
CXCR5-PE-Cy7
Singlets
Lymphocytes
Live cells
CD8 v CD4
IFNγ + CD8 +
SSC singlets 95.1
Lymphocytes 72.0
live cells 91.3
CD8+ 24.1
CD4+ 33.9
IFNg+ (v CD44) 0.41
L/D-Near-IR
IL-21 + CD4 +
IFNγ + CD4 +
IL-21+ (v CD44) 0.14
IFNg+ (v CD44) 0.82
IL-21-Fc fusion protein
IFNγ-APC
CD44-Brilliant Violet 785
Singlets
Lymphocytes
Dump −
B cells
GC B cells
SSC singlets 85.0
Lymphocytes 66.2
dump- 94.2
B220+ CD3- 29.0
PNA+Fas+ 8.32
L/D Near-IR + F4/80-APC-Cy7 + NK1.1-APC-Cy7
B220-AlexaFluor 700
CD3-Brilliant Violet 650
Fas-APC
PNA-FITC

Supplementary Figure 16. Representative flow cytometry plots and gating strategy (a-d) for the results presented in the Figure 7c.

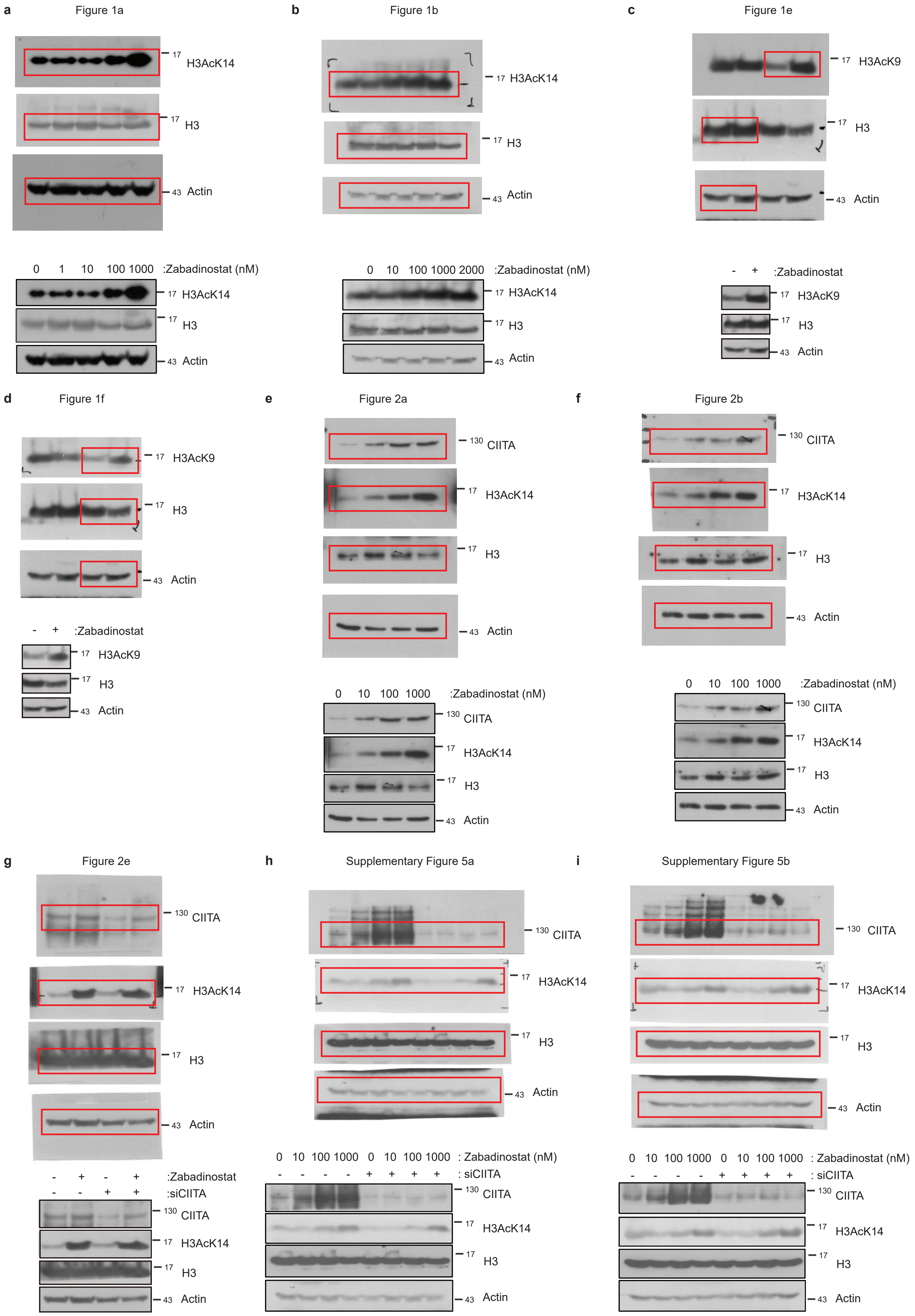
a
Figure 1a
17 H3AcK14
17 H3
43 Actin
0 1 10 100 1000 :Zabadinostat (nM)
17 H3AcK14
17 H3
43 Actin
b
Figure 1b
17 H3AcK14
17 H3
43 Actin
0 10 100 1000 2000 :Zabadinostat (nM)
17 H3AcK14
17 H3
43 Actin
c
Figure 1e
17 H3AcK9
17 H3
43 Actin
- + :Zabadinostat
17 H3AcK9
17 H3
43 Actin
d
Figure 1f
17 H3AcK9
17 H3
43 Actin
- + :Zabadinostat
17 H3AcK9
17 H3
43 Actin
e
Figure 2a
130 CIITA
17 H3AcK14
17 H3
43 Actin
0 10 100 1000 :Zabadinostat (nM)
130 CIITA
17 H3AcK14
17 H3
43 Actin
f
Figure 2b
130 CIITA
17 H3AcK14
17 H3
43 Actin
0 10 100 1000 :Zabadinostat (nM)
130 CIITA
17 H3AcK14
17 H3
43 Actin
g
Figure 2e
130 CIITA
17 H3AcK14
17 H3
43 Actin
- + - + :Zabadinostat
- - + + :siCIITA
130 CIITA
17 H3AcK14
17 H3
43 Actin
h
Supplementary Figure 5a
130 CIITA
17 H3AcK14
17 H3
43 Actin
0 10 100 1000 0 10 100 1000 : Zabadinostat (nM)
- - - - + + + + : siCIITA
130 CIITA
17 H3AcK14
17 H3
43 Actin
i
Supplementary Figure 5b
130 CIITA
17 H3AcK14
17 H3
43 Actin
0 10 100 1000 0 10 100 1000 : Zabadinostat (nM)
- - - - + + + + : siCIITA
130 CIITA
17 H3AcK14
17 H3
43 Actin

Supplementary Figure 17. Uncropped versions of immunoblots used in the Figures 1a, 1b, 1e, 1f, 2a, 2b, 2e, Supplementary Figure 5a, and 5b.

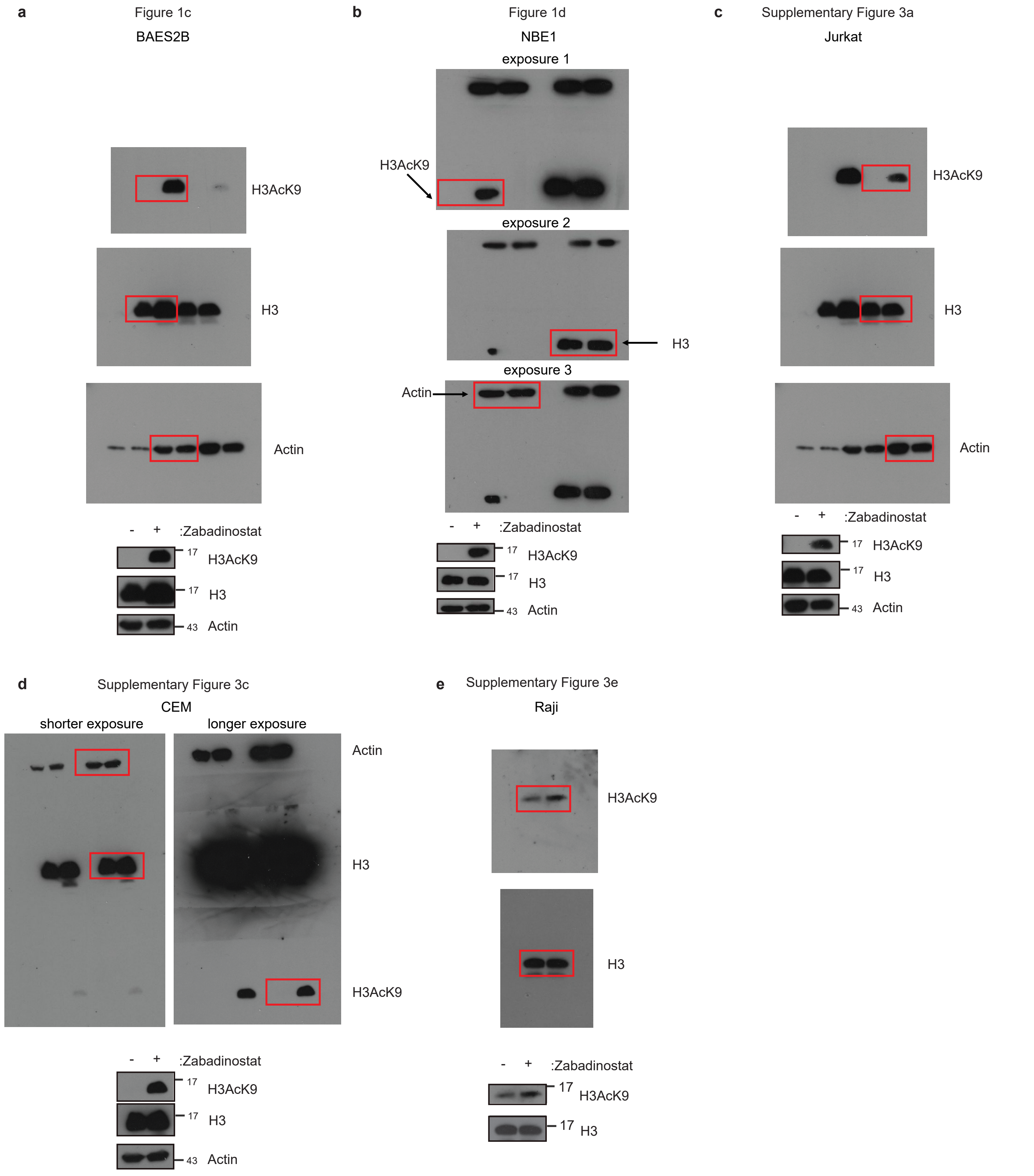
a
Figure 1c
BAES2B
H3AcK9
H3
Actin
- + :Zabadinostat
17 H3AcK9
17 H3
43 Actin
b
Figure 1d
NBE1
exposure 1
H3AcK9
exposure 2
H3
exposure 3
Actin
- + :Zabadinostat
17 H3AcK9
17 H3
43 Actin
c
Supplementary Figure 3a
Jurkat
H3AcK9
H3
Actin
- + :Zabadinostat
17 H3AcK9
17 H3
43 Actin
d
Supplementary Figure 3c
CEM
shorter exposure
longer exposure
Actin
H3
H3AcK9
- + :Zabadinostat
17 H3AcK9
17 H3
43 Actin
e
Supplementary Figure 3e
Raji
H3AcK9
H3
- + :Zabadinostat
17 H3AcK9
17 H3

Supplementary Figure 18. Uncropped versions of immunoblots used in the Figures 1c, 1d, Supplementary Figure 3a, 3c, and 3e.
