# Supplementary material for: The HDAC inhibitor zabadinostat is a systemic regulator of adaptive immunity
Source: Commun Biol. 2023 Jan 26;6:102. doi: 10.1038/s42003-023-04485-y (PMC9878486; doi:10.1038/s42003-023-04485-y)
Supplement: Supplementary file 4 — Reporting Summary [file 42003_2023_4485_MOESM4_ESM.pdf]

nature portfolio

Corresponding author(s): COMMSBIO-22-1040C

Last updated by author(s): Jan 4, 2023

Reporting Summary

Nature Portfolio wishes to improve the reproducibility of the work that we publish. This form provides structure for consistency and transparency in reporting. For further information on Nature Portfolio policies, see our Editorial Policies and the Editorial Policy Checklist.

Statistics

For all statistical analyses, confirm that the following items are present in the figure legend, table legend, main text, or Methods section.

n/a	Confirmed	
☐	☒	The exact sample size (*n*) for each experimental group/condition, given as a discrete number and unit of measurement
☐	☒	A statement on whether measurements were taken from distinct samples or whether the same sample was measured repeatedly
☐	☒	The statistical test(s) used AND whether they are one- or two-sided<br>*Only common tests should be described solely by name; describe more complex techniques in the Methods section.*
☒	☐	A description of all covariates tested
☒	☐	A description of any assumptions or corrections, such as tests of normality and adjustment for multiple comparisons
☐	☒	A full description of the statistical parameters including central tendency (e.g. means) or other basic estimates (e.g. regression coefficient) AND variation (e.g. standard deviation) or associated estimates of uncertainty (e.g. confidence intervals)
☐	☒	For null hypothesis testing, the test statistic (e.g. *F*, *t*, *r*) with confidence intervals, effect sizes, degrees of freedom and *P* value noted<br>*Give P values as exact values whenever suitable.*
☒	☐	For Bayesian analysis, information on the choice of priors and Markov chain Monte Carlo settings
☒	☐	For hierarchical and complex designs, identification of the appropriate level for tests and full reporting of outcomes
☒	☐	Estimates of effect sizes (e.g. Cohen's *d*, Pearson's *r*), indicating how they were calculated

Our web collection on statistics for biologists contains articles on many of the points above.

Software and code

Policy information about availability of computer code

Data collection	Data was collected exclusively using commercial softwares (AriaMX real-time qPCR instrument (Aria MX, Agilent); LICOR C-Digit (LI-COR Biosciences, Lincoln, NE, USA; TECAN sunrise plate reader (Tecan, Switzerland); BD LSRII flow cytometer (Oxford, UK); Attune NXT flow cytometer (Thermo Fisher, Waltham, MA, USA); Leica DM2500 optical microscope), BD Accuri C6, BD Fortessa
Data analysis	Data was analysed exclusively using commercial softwares (Aria MX software; ImageJ software (National Institutes of Health, USA); Graphpad prism 8 Software (GraphPad Software); FlowJo software, BD Accuri C6 software

For manuscripts utilizing custom algorithms or software that are central to the research but not yet described in published literature, software must be made available to editors and reviewers. We strongly encourage code deposition in a community repository (e.g. GitHub). See the Nature Portfolio guidelines for submitting code & software for further information.

Data

Policy information about availability of data

All manuscripts must include a data availability statement. This statement should provide the following information, where applicable:

- Accession codes, unique identifiers, or web links for publicly available datasets
- A description of any restrictions on data availability
- For clinical datasets or third party data, please ensure that the statement adheres to our policy

All data generated or analysed during this study are included in this published article (and its supplementary information files). Materials generated in this study are available from the corresponding authors upon reasonable request.

Field-specific reporting

Please select the one below that is the best fit for your research. If you are not sure, read the appropriate sections before making your selection.

☒ Life sciences ☐ Behavioural & social sciences ☐ Ecological, evolutionary & environmental sciences

For a reference copy of the document with all sections, see nature.com/documents/nr-reporting-summary-flat.pdf

Life sciences study design

All studies must disclose on these points even when the disclosure is negative.

Sample size	Cells: No sample size was predetermined. Sample sizes were chosen according to the standard generally accepted in the field (at least three biological replicates)<br>Mouse experiments: Size of the groups was determined based on power calculation to reach >80% power for the outputs required, and previous experience
Data exclusions	No data were excluded
Replication	Cells: Each experiment was reproduced at least three times with similar results. All attempts at replication were successful.<br>Mouse experiments: All experiments were performed in the appropriate number of mice according to power calculation.
Randomization	In mouse exeriments animals were randomly assigned.
Blinding	Cells: No blinding required.<br>Mouse experiments: the scientist performing following key laboratory analyses (flow cytometry, ELISA) is not involved in the mouse handling and is blinded to the experimental protocols and animal allocation.

Reporting for specific materials, systems and methods

We require information from authors about some types of materials, experimental systems and methods used in many studies. Here, indicate whether each material, system or method listed is relevant to your study. If you are not sure if a list item applies to your research, read the appropriate section before selecting a response.

Materials & experimental systems

n/a	Involved in the study	
☐	☒	Antibodies
☐	☒	Eukaryotic cell lines
☒	☐	Palaeontology and archaeology
☐	☒	Animals and other organisms
☒	☐	Human research participants
☒	☐	Clinical data
☒	☐	Dual use research of concern

Methods

n/a	Involved in the study	
☒	☐	ChIP-seq
☐	☒	Flow cytometry
☒	☐	MRI-based neuroimaging

Antibodies

Antibodies used	anti-Histone H3 [ab1791, Abcam], anti-H3AcK9 (ab10812, Abcam), anti-H3AcK14 (#7627, Cell Signaling, Danvers, MA, USA), anti-β-Actin (#3700, Cell Signaling), anti-CIITA (TA319682, Origene, Rockville, MD, USA), anti-CD40 (anti-CD40, 2bScientific), CD3 (100210, Biolegend, San Diego, CA, USA), CD45 (103132, Biolegend), and CD4 (558107, BD Biosciences, New York, USA), IFNγ (554413, BD Biosciences), TNFα (12-7321-82, eBiosciences, Waltham, MA, USA), LEGENDplex MU Th Cytokine Panel (12-plex) VbP V03 (741044, Biolegend), anti-CD8 (ab203035; Abcam), anti-CD4 (ab183685, Abcam), CD3 (561826, BD Pharmingen), CD8 (553032, BD Pharmingen), CD4 (557308, BD Pharmingen), CD20 (150409, Biolegend), CD69 (557392, BD Pharmingen), HLA-ABC (560965, BD Pharmingen), HLA- DR, DP, DQ (562008, BD Pharmingen), CD11c (557400, BD Pharmingen), CD86 (561964, BD Pharmingen); anti-CXCR5 antibody (clone L138D7, BioLegend), Streptavidin PE-Cy7 (Biologend), anti-PD-1 APC (clone RMP1-30, Biologend), anti-CD8 AlexaFluor 700 (clone 53-6.7, Biologend); anti-B220 APC-Cy7 (clone RA3-6B2, Biologend), anti-F4/80 APC-Cy7 (clone BM8, Biologend), anti-CD4 Brilliant Violet 650 (clone RM4-5, Biologend), anti-CD44 Brilliant Violet 785 (clone IM7, Biologend), and Near-IR Live/Dead dye (ThermoFisher), Brefeldin A (BioLegend), IL-21R-Fc fusion protein (R&D Systems), Fc-PE (AB_2337681,Jackson ImmunoResearch), anti-IFNγ APC (clone XMG1.2, Biologend), anti-CD8 AlexaFluor 700 (clone 53-6.7, Biologend), anti-CD4 Brilliant Violet 650 (clone RM4-5, Biologend), anti-CD44 Brilliant Violet 785 (clone IM7, Biologend), TruStain FcX (clone 93, Biolegend), Peanut agglutinin (PNA) FITC (Vector Labs), anti-Fas (clone 15A7, eBioscience), anti-B220 AlexaFluor 700 (clone RA3-6B2, BioLegend), anti-F4/80 APC-Cy7 (clone BM8, BioLegend), anti-NK1.1 APC-Cy7 (clone PK136, BioLegend), anti-CD3 Brilliant Violet 650 (clone 17A2, BioLegend), Alexa Fluor™ 594 (a21207, Life Technologies), H2 class I (566776, BD Pharmingen).

Validation	All antibodies are commercially available and are validated by the manufacturer (which can found via specific Brand and catalog numbers provided above) and in previous publications

Eukaryotic cell lines

Policy information about cell lines

Cell line source(s)	- ATCC - HCT116 (CCL-247) and BEAS-2B (CRL-9609)<br>- NBE1 cell line was donated by Prof. Anderson Ryan lab (Oxford, UK)<br>- Jurkat cell line donated by Prof. Eric O'Neill (Department of Oncology, University of Oxford)<br>- CEM and Raji cell lines donated by Prof. Quentin Sattentau (Sir William Dunn School of Pathology, University of Oxford)
Authentication	Authentication was certified by ATCC. NBE1 cell line was authenticated by Prof. Ryan. Jurkat cell line was authenticated by Prof. O'Neill. CEM and Raji cell lines were authenticated by Prof. Sattentau.
Mycoplasma contamination	All cell line were tested negative for mycoplasma contamination
Commonly misidentified lines (See ICLAC register)	No commonly misidentified cell lines were used.

Animals and other organisms

Policy information about studies involving animals; ARRIVE guidelines recommended for reporting animal research

Laboratory animals	Balb/c female mice at age 6-8 weeks<br>C57BL/6 female mice at age 26 weeks
Wild animals	No wild animals included.
Field-collected samples	No field-collected samples included.
Ethics oversight	Charles River Discovery experiments and protocols were approved by the animal welfare body at Charles River Discovery Research Services Germany (where each experiment was performed) and the local authorities, and were conducted according to all applicable international, national and local laws and guidelines.<br><br>Oxford University experiments were performed according to UK Home Office regulations and after review and approval by the local ethical review board at the University of Oxford. Mice were cared for in accordance with institutional guidelines.

Note that full information on the approval of the study protocol must also be provided in the manuscript.

Flow Cytometry

Plots

Confirm that:

☒ The axis labels state the marker and fluorochrome used (e.g. CD4-FITC).

☒ The axis scales are clearly visible. Include numbers along axes only for bottom left plot of group (a 'group' is an analysis of identical markers).

☒ All plots are contour plots with outliers or pseudocolor plots.

☒ A numerical value for number of cells or percentage (with statistics) is provided.

Methodology

Sample preparation	FACS-Buffer:<br>PBS containing 0.1 % BSA<br><br>Life/Dead Stain:<br>Aqua Zombie (BioLegend, #423102) 1:100 in PBS<br><br>Fc block:<br>CD16/32 purified (clone 2.4G2, BD Biosciences, #553142), 0.5 mg/ml<br><br>ACK-Lysis-Buffer (1x) 1L (0.15M NH4Cl, 10mM KHCO3, 0.1mM EDTA):<br>• 8.3g NH4Cl<br>• 1.0g KHCO3<br>• 200µl 0.5M EDTA<br>• Fill up to 1 liter with H2O Millipore<br><br>Fixation/ Permeabilization:<br>Intracellular Fixation & Permeabilization Buffer Set (eBioscience™, #88-8824-00)

Protein secretion inhibition:
BD GolgiPlugTM protein transport inhibitor containing BrefeldinA (BD Biosciences, #555029)
BD GolgiStop protein transport inhibitor containing Monensin from BD Biosciences (#554724)
Use 6 µl GolgiStop + 4 µl GolgiPlug per ml as 4x concentration.

Ex vivo stimulation:
PMA (Stemcell, #74042), final conc. 10 ng/ml (=1x)
Ionomycin (Stemcell, #73722), final conc. 1 µg/ml (=1x)

Flow Cytometry Protocol

Preparation of spleen single cell suspensions:
- Harvest spleen and transfer into tube containing precooled sterile PBS, store on ice until further processing
- Smash spleen(s) over a 100 µm cell strainer (BD) with the stamp of a syringe into a 50 ml tube
- Rinse cell strainer with 10 ml cold PBS
- Centrifuge at 300xg, 5min, 4°C
- Discard supernatant and resuspend cells by tapping
- Add 4 ml 1x ice-cold ACK-Lysis Buffer per 50 ml tube
- Lyse for 50 sec at RT processing up to 4 tubes in one "batch"
- Fill tubes up to 50 ml with ice-cold PBS
- Centrifuge at 300xg, 5min, 4°C, discard SN by decanting
- Resuspend pellet by tapping, add appropriate volume of ice-cold PBS
- Resuspend thoroughly by pipetting and remove debris
- Pool cells from animals of same group
- Take sample for counting
- Centrifuge at 300xg, 5 min, 4°C
- Remove supernatant by decanting, resuspend pellet by tapping
- Resuspend cells at 2x106 (2 million) cells/100 µl in medium (RPMI1640 with Gln, #FG 1385, Biochrom AG, RT supplemented with 10% FCS

Ex vivo stimulation of spleen single cell suspensions:

- Transfer 100 µl of cell suspension (2x106 (2 million) cells/100 µl) into each well of a round bottom 96 well plate
- Add 50 µl of medium containing 4x concentrated treatments as specified for the respective study (here see paragraph 4).
- Mix by pipetting up and down 3 times with 100 µl (multichannel pipet).
- Incubate for 2h, 37°C, 5% CO2
- Add 50 µl of medium containing 4x concentrated GolgiStop + GolgiPlug
- Mix by pipetting up and down 3 times with 100 µl (multichannel pipet).
- Incubate for another 4h, 37°C, 5% CO2

Surface staining:
- Centrifuge plate at 700xg for 3 min at RT, remove SN by flicking
- Add 200 µl FACS Buffer, centrifuge at 700xg for 3 min at RT, remove SN by flicking
- Add 10 µl Fc-Block (diluted 1:100 in FACS-Buffer) per well
- Incubate 5 min at RT
- Add 50 µl AquaZombie live dead stain (1:100) diluted in PBS, containing respective amount of directly conjugated primary antibody for detection of surface antigen(s) to the cells and incubate 30 min at 4°C in the dark
- Wash with 200 µl cold FACS buffer by centrifugation, 700xg for 3 min at 4°C, repeat washing step

Intracellular staining:
Intracellular Fixation & Permeabilization Buffer Set (eBioscience™, #88-8824-00), all incubation steps (duration, temperature) according to manufacturer's instructions

- After washing, discard the supernatant by flicking and pulse vortex the sample to completely dissociate the pellet
- Fix the cells by adding 100 µL of IC Fixation Buffer and pulse vortex to mix
- Incubate 40 min at RT, protect from light
- Centrifuge at 400xg for 5 min at RT, remove supernatant by pipetting, resuspend cells in 200 µl of 1X Permeabilization Buffer and centrifuge at 400xg for 5 min at RT, discard the supernatant by pipetting
- Wash again with 200 µl of 1X Permeabilization Buffer and centrifuge at 400xg for 5 min at RT, discard the supernatant by pipetting
- Resuspend the cell pellet in 50 µL of 1X Permeabilization Buffer containing the recommended amount of directly conjugated primary antibody for detection of intracellular antigen(s) to the cells and incubate for 40 min at RT, protect from light
- Add 200 µl of 1X Permeabilization Buffer and centrifuge at 400xg for 5 min at RT, discard supernatant by pipetting. Repeat once again.
- Resuspend stained cells thoroughly in 200 µl cold FACS Buffer/well and transfer into deep well plate
- Inspect wells visually for remaining cell pellets, add again 200 µl of cold FACS Buffer/well, resuspend and transfer into same deep well plate (final volume/ well for FC analysis = 400 µl)
- Analyze on Attune NXT Acoustic Focusing Cytometer

For C57BL/6 immunogenicity experiment with I8V tetramers, please see the publications:
Colston, J. M. et al. Divergent memory responses driven by adenoviral vectors are impacted by epitope competition. Eur J Immunol 49, 1356-1363, doi:10.1002/eji.201948143 (2019)
Lee, L. N. et al. Adenoviral vaccine induction of CD8+ T cell memory inflation: Impact of co-infection and infection order. PLoS Pathog 13, e1006782, doi:10.1371/journal.ppat.1006782 (2017).

MHC class I and II expression analysis
NBE1, BEAS-2B, Jurkat, CEM, and Raji cells were stained with HLA-ABC (560965, BD Pharmingen, 1:100) and HLA- DR, DP, DQ (562008, BD Pharmingen, 1:100) antibodies. Bone marrow-derived DCs dendritic cells were harvested and stained with CD11c, CD86 and/or H2- I-A/I-E (107613, Biolegend, 1:100) antibodies. After 30 minutes incubation at room temperature, cells were analysed by flow cytometry (BD Accuri C6, Becton Dickinson). Additionally, the level of acetylation and histone H3 was assessed after fixation (4% formaldehyde solution for 15 min, room temperature) and permeabilisation (0.5% Triton X-100 solution for 15 min, room temperature) of the DCs cells with H3 (Abcam, 1:500), and H3AcK14 (Cell Signaling, 1:500) antibodies.

T cell and B cell activation experiment
The splenocytes were resuspended in 1 ml ACK lysis buffer (Lonza) for 3-5 min to lyse the red blood cells, then stopped with 20 ml PBS, followed by centrifugation at 1500 rpm, 5 min at room temperature. The splenocyte pellet was resuspended in RPMI medium, splenocytes were seeded and treated with 1 µM zZabadinostat or PHA-L (11249738001, Merck) for 48h. Activation of CD8, CD4, and B cells was evaluated by flow cytometry (BD Accuri C6). The following antibodies were used: CD3 (561826, BD Pharmingen, 1:100), CD8 (553032, BD Pharmingen, 1:100), CD4 (557308, BD Pharmingen, 1:100), CD20 (150409, Biolegend, 1:100), CD69 (557392, BD Pharmingen, 1:100).

TFH cell phenotyping
Identification of CXCR5+PD-1+ TFH cells was performed as previously described, with modification. Splenocytes (200 µl) were plated in a U-bottom 96-well plate and washed one time with FACS buffer (PBS + 1 mM EDTA + 0.05% BSA). Cells were then stained for 30 min at 4C with biotinylated anti-CXCR5 antibody (clone L138D7, 1:100 dilution, BioLegend). Cells were washed two times with FACS buffer. The remaining staining cocktail was then added: Streptavidin PE-Cy7 (1:1000), anti-PD-1 APC (clone RMP1-30, 1:50), anti-CD8 AlexaFluor 700 (clone 53-6.7, 1:100), anti-B220 APC-Cy7 (clone RA3-6B2, 1:100), anti-F4/80 APC-Cy7 (clone BM8, 1:100), anti-CD4 Brilliant Violet 650 (clone RM4-5, 1:100), anti-CD44 Brilliant Violet 785 (clone IM7, 1:100), and Near-IR Live/Dead dye (1:400). All reagents from BioLegend except viability dye which was from ThermoFisher. After 30 min at 4 ℃, cells were washed two times with FACS buffer. Cells were fixed for 20 min at 4 ℃ using the Cytofix/Cytoperm kit (BD Biosciences). Following two final washes in FACS buffer, samples were stored at 4 ℃ until data acquisition on a BD Fortessa flow cytometer. Data analysis was performed using FlowJo v. 10.8.1.

Intracellular cytokine staining
Intracellular cytokine staining was performed as previously described. Briefly, splenocytes (100 µl) were plated in a U-bottom 96-well plate. Cells were stimulated with 1 µg/ml of overlapping SARS-CoV-2 spike peptide pool covering the entire protein (15mers overlapping by 11; JPT peptides). Brefeldin A (1:1000, BioLegend) was added at the time of stimulation and cells were cultured at 37℃, 5% CO2 for 5 h. After stimulation, cells were washed one time with FACS buffer (PBS + 1 mM EDTA + 0.05% BSA). Cells were stained for 10 min at 4℃ with Near-IR Live/Dead dye (1:400, ThermoFisher). Cells were washed twice with FACS buffer. Cells were fixed for 20 min at 4℃ using the Cytofix/Cytoperm kit (BD Biosciences), followed by two washes with 1× Perm/Wash buffer (BD Biosciences). Staining for IL-21 was performed using an mouse IL-21R-Fc fusion protein (1:50, R&D Systems) for 30 min at 4℃. After two washes with 1× Perm/Wash buffer, cells were stained with anti-human Fc-PE (AB_2337681, 1:50, Jackson ImmunoResearch) for 30 min at 4 ℃. Cells were washed two times with 1× Perm/Wash buffer. The final round of staining was for 30 min at 4℃ comprised a cocktail of: anti-IFNγ APC (clone XMG1.2, 1:100), anti-CD8 AlexaFluor 700 (clone 53-6.7, 1:100), anti-CD4 Brilliant Violet 650 (clone RM4-5, 1:100), and anti-CD44 Brilliant Violet 785 (clone IM7, 1:100). All antibodies from BioLegend. Two final washes with 1× Perm/Wash buffer were performed before storage of the cells at 4 ℃ in FACS buffer for acquisition on a BD Fortessa flow cytometer. Data analysis was performed using FlowJo v. 10.8.1.

GC B cell phenotyping
Identification of Fas+PNA+ GC B cells was performed as previously described, with slight modification. Splenocytes (200 µl) were plated in a U-bottom 96-well plate and washed one time with FACS buffer (PBS + 1 mM EDTA + 0.05% BSA). Cells were then stained for 30 min at 4℃ with mouse TruStain FcX (clone 93, 1:50, BioLegend). Cells were washed two times with FACS buffer. The surface cocktail was then added: Peanut agglutinin (PNA) FITC (1:5000, Vector Labs), anti-Fas (clone 15A7, 1:50, eBioscience), anti-B220 AlexaFluor 700 (clone RA3-6B2, 1:100, BioLegend), anti-F4/80 APC-Cy7 (clone BM8, 1:100, BioLegend), anti-NK1.1 APC-Cy7 (clone PK136, 1:100, BioLegend), anti-CD3 Brilliant Violet 650 (clone 17A2, 1:100, BioLegend), and Near-IR Live/Dead dye (1:400 dilution, ThermoFisher). After 30 min at 4℃, cells were washed two times with FACS buffer. Cells were fixed for 20 min at 4℃ using the Cytofix/Cytoperm kit (BD Biosciences). Following two final washes in FACS buffer, samples were stored at 4℃ until data acquisition on a BD Fortessa flow cytometer. Data analysis was performed using FlowJo v. 10.8.1.

For more details, please see:
1. Provine, N. M., A. Amini, L. C. Garner, A. J. Spencer, C. Dold, C. Hutchings, L. S. Reyes, M. E. B. FitzPatrick, S. Chinnakannan, B. Oguti, M. Raymond, M. Ulaszewska, F. Troise, H. Sharpe, S. B. Morgan, T. S. C. Hinks, T. Lambe, S. Capone, A. Folgori, E. Barnes, C. S. Rollier, A. J. Pollard, and P. Klenerman. 2021. MAIT cell activation augments adenovirus vector vaccine immunogenicity. Science 371: 521–526.
2. Johnston, R. J., Y. S. Choi, J. A. Diamond, J. A. Yang, and S. Crotty. 2012. STAT5 is a potent negative regulator of TFH cell differentiation. J Exp Med 209: 243–250.
3. Provine, N. M., A. Badamchi-Zadeh, C. A. Bricault, P. Penaloza-Macmaster, R. A. Larocca, E. N. Borducchi, M. S. Seaman, and D. H. Barouch. 2016. Transient CD4+ T Cell Depletion Results in Delayed Development of Functional Vaccine-Elicited Antibody Responses. Journal of Virology 90: 4278–4288.

Instrument	BD LSRII flow cytometer (Oxford, UK); Attune NXT flow cytometer (Thermo Fisher, Waltham, MA, USA), BD Accuri C6 (BD), BD Fortessa
Software	Data were analysed using FlowJo software or BD Accuri C6 software

Cell population abundance

Abundance of cell population of interest was determined by appropriate negative control and the purity of sorted populations was assessed by post-sort analysis.

Gating strategy

- FSC-H/FSC-A; FSC-H/SSC-H; count/LD dye; count/CD45; count/CD3; CD4/TNFa and CD4/IFNγ
- FSC-H/FSC-A; FSC-H/SSC-H; count/LD dye; CD44+ CD62L- CD8+; I8V-tet+ CD8+; I8V-tet+ CD8+IFNγ+; I8V-tet+ CD8+TNFa+; I8V-tet+ CD8+IL-2+
- SSC-A/FSC-A; HLA-ABC+ or HLA-DR,DP,DQ+
- SSC-A/FSC-A; CD11c+/CD86+ or CD11c+/H2-A/E+ or CD11c+H3K14Ac or CD11c+H3+
- SSC-A/FSC-A; CD3+/CD4+/CD69+ or CD3+/CD8+/CD69+ or CD3-/CD20+/CD69+
- SSC-A/FSC-A; FL2-A and FL4-A
- SSC-A/SSCH; SSC-A/FSC-A; SSC-A/L/D + F4/80 + B220; CD4/CD8; PD-1/CXCR5
- SSC-A/SSCH; SSC-A/FSC-A; SSC-A/LD; CD4/CD8; CD4/IFNγ/CD44 or CD8/IFNγ/CD44 or CD4/IL-21/CD44
- SSC-A/SSCH; SSC-A/FSC-A; SSC-A/L/D + F4/80 + NK1.1; B220/CD3; Fas/PNA

☒ Tick this box to confirm that a figure exemplifying the gating strategy is provided in the Supplementary Information.
